# Clinical and pulmonary function analysis in long-COVID revealed that long-term pulmonary dysfunction is associated with vascular inflammation pathways and metabolic syndrome

**DOI:** 10.3389/fmed.2023.1271863

**Published:** 2023-10-06

**Authors:** Sergio Sanhueza, Mabel A. Vidal, Mauricio A. Hernandez, Mario E. Henriquez-Beltran, Camilo Cabrera, Romina Quiroga, Bárbara E. Antilef, Kevin P. Aguilar, Daniela A. Castillo, Faryd J. Llerena, Marco Fraga Figueroa, Mauricio Nazal, Eritson Castro, Paola Lagos, Alexa Moreno, Jaime J. Lastra, Jorge Gajardo, Pamela Garcés, Benilde Riffo, Jorge Buchert, Rocío Sanhueza, Valeska Ormazába, Pablo Saldivia, Cristian Vargas, Guillermo Nourdin, Elard Koch, Felipe A. Zuñiga, Liliana Lamperti, Paula Bustos, Enrique Guzmán-Gutiérrez, Claudio A. Tapia, Luciano Ferrada, Gustavo Cerda, Ute Woehlbier, Erick Riquelme, Maria-Isabel Yuseff, Braulio A. Muñoz Ramirez, Giovanna Lombardi, David De Gonzalo-Calvo, Carlos Salomon, Ricardo A. Verdugo, Luis A. Quiñones, Alicia Colombo, Maria I. Barría, Gonzalo Labarca, Estefania Nova-Lamperti

**Affiliations:** ^1^Molecular and Translational Immunology Laboratory, Department of Clinical Biochemistry and Immunology, Pharmacy Faculty, University of Concepción, Concepción, Chile; ^2^Facultad de Ingeniería, Diseño y Arquitectura, Universidad San Sebastián, Concepción, Chile; ^3^Division of Biotechnology, MELISA Institute, San Pedro de la Paz, Chile; ^4^Núcleo de Investigación en Ciencias de la Salud, Universidad Adventista de Chile, Chillán, Chile; ^5^Kinesiology School, Escuela de Kinesiología, Facultad de Salud, Universidad Santo Tomás, Los Ángeles, Chile; ^6^Translational Research in Respiratory Medicine, University Hospital Arnau de Vilanova and Santa Maria, IRBLleida, Lleida, Spain; ^7^Internal Medicine Department, Hospital Guillermo Grant Benavente and Medicine Faculty, University of Concepción, Concepción, Chile; ^8^PreveGen Laboratory, Concepción, Chile; ^9^Department of Pharmacology, Faculty of Biological Sciences, University of Concepción, Concepción, Chile; ^10^CMA Bío-Bío - Advanced Microscopy Center, University of Concepción, Concepción, Chile; ^11^Center for Integrative Biology, Faculty of Sciences, Universidad Mayor, Santiago, Chile; ^12^Faculty of Medicine, Pontifical Catholic University of Chile, Santiago, Chile; ^13^Facultad de Ciencias Biológicas, Pontificia Universidad Católica de Chile, Santiago, Chile; ^14^Department of Pharmacology and Toxicology, School of Medicine, Indiana University Bloomington, Bloomington, IN, United States; ^15^Peter Gorer Department of Immunobiology, School of Immunology and Microbial Sciences, Faculty of Life Sciences and Medicine, King’s College London, London, United Kingdom; ^16^CIBER of Respiratory Diseases (CIBERES), Institute of Health Carlos III, Madrid, Spain; ^17^Exosome Biology Laboratory, Centre for Clinical Diagnostics, UQ Centre for Clinical Research, Royal Brisbane and Women’s Hospital, Medicine and Biomedical Science Faculty, The University of Queensland, Brisbane, QLD, Australia; ^18^Instituto de Investigación Interdisciplinaria y Escuela de Medicina, Universidad de Talca, Talca, Chile; ^19^Department of Basic-Clinical Oncology, Faculty of Medicine, University of Chile, Santiago, Chile; ^20^Department of Pharmaceutical Sciences and Technology, Faculty of Chemical and Pharmaceutical Sciences, University of Chile, Santiago, Chile; ^21^Latin American Network for Implementation and Validation of Clinical Pharmacogenomics Guidelines (RELIVAF-CYTED), Santiago, Chile; ^22^Servicio de Anatomía Patológica, Hospital Clínico, Universidad de Chile, Santiago, Chile; ^23^Facultad de Medicina y Ciencia, Universidad San Sebastián, Puerto Montt, Chile; ^24^Internal Medicine, Complejo Asistencial Dr. Víctor Ríos Ruiz, Los Ángeles, Chile; ^25^Division of Sleep and Circadian Disorders, Brigham and Women’s Hospital, Harvard Medical School, Boston, MA, United States

**Keywords:** COVID-19, pulmonary dysfunction, sequelae, chemokines, vascular inflammation, metabolic syndrome

## Abstract

**Introduction:**

Long-term pulmonary dysfunction (L-TPD) is one of the most critical manifestations of long-COVID. This lung affection has been associated with disease severity during the acute phase and the presence of previous comorbidities, however, the clinical manifestations, the concomitant consequences and the molecular pathways supporting this clinical condition remain unknown. The aim of this study was to identify and characterize L-TPD in patients with long-COVID and elucidate the main pathways and long-term consequences attributed to this condition by analyzing clinical parameters and functional tests supported by machine learning and serum proteome profiling.

**Methods:**

Patients with L-TPD were classified according to the results of their computer-tomography (CT) scan and diffusing capacity of the lungs for carbon monoxide adjusted for hemoglobin (DLCOc) tests at 4 and 12-months post-infection.

**Results:**

Regarding the acute phase, our data showed that L-TPD was favored in elderly patients with hypertension or insulin resistance, supported by pathways associated with vascular inflammation and chemotaxis of phagocytes, according to computer proteomics. Then, at 4-months post-infection, clinical and functional tests revealed that L-TPD patients exhibited a restrictive lung condition, impaired aerobic capacity and reduced muscular strength. At this time point, high circulating levels of platelets and CXCL9, and an inhibited FCgamma-receptor-mediated-phagocytosis due to reduced FcγRIII (CD16) expression in CD14+ monocytes was observed in patients with L-TPD. Finally, 1-year post infection, patients with L-TPD worsened metabolic syndrome and augmented body mass index in comparison with other patient groups.

**Discussion:**

Overall, our data demonstrated that CT scan and DLCOc identified patients with L-TPD after COVID-19. This condition was associated with vascular inflammation and impair phagocytosis of virus-antibody immune complexes by reduced FcγRIII expression. In addition, we conclude that COVID-19 survivors required a personalized follow-up and adequate intervention to reduce long-term sequelae and the appearance of further metabolic diseases.

## 1. Introduction

Severe acute respiratory syndrome coronavirus-2 (SARS-CoV-2) is the etiology agent of coronavirus disease 2019 (COVID-19), which has become the largest pandemic disease in the last century (1, 2). This infectious disease normally presents mild symptoms, but it can progress from moderate to severe, mainly, but not exclusively, in elderly patients with comorbidities such as hypertension, type 2 diabetes mellitus (T2DM), and obesity (3, 4). Severe COVID-19 is characterized by acute respiratory distress syndrome (5–7) due to an exacerbated inflammatory response (8, 9) and cytokine storm (10). In addition, cells from the innate response such as neutrophils and monocytes are augmented in circulation (11, 12), whereas cells from the adaptive immune response such as lymphocytes have been found reduced (13, 14). Several reports have shown that pathways such as microvascular injury (15–17), hyperinflammation by immune system dysregulation (18–20), and thrombosis (21) are associated with COVID-19 severity during the acute phase, which support lung damage and the requirement of oxygen support by non-invasive or invasive mechanical ventilation.

Coronavirus disease 2019 patients exhibited sustained and diverse sequelae after acute disease, and more recently several researchers have used the terminology of post-acute COVID-19, post-COVID-19 syndrome, or long-COVID-19 to define this condition (22–24). However, it is relevant to understand the timeline, the persistence, and the diversity of these sequelae as they are not uniform in recovered patients (25, 26). A recent review has defined post-acute COVID-19 as between 4 and 12 weeks after acute COVID-19, whereas post-COVID-19 syndrome was defined as lasting beyond 12 weeks after the onset of acute COVID-19 and as not attributable to other possible causes (27). However, the definition of this condition is still evolving according to studies revealing new sequelae or long-term physical conditions. The main sequelae described up to date include complications of the pulmonary and cardiovascular system, hematological parameters, neuropsychiatry, and renal function (25, 28–32).

Several pulmonary manifestations have been reported among COVID-19 survivors (25, 28, 33). For example, alteration in the computed tomography (CT) scan after infection has been associated with the requirement of invasive mechanic ventilation during the acute phase of the disease (34–36), whereas a reduction in the diffusion capacity for carbon monoxide (DLCO) is one of the most reported lung function impairments 6-months after COVID-19 (25, 28, 33). In addition, severe acute COVID-19 has been associated with a higher risk of long-term pulmonary sequelae, including pulmonary structural abnormalities and impaired O_2_ diffusion (25, 28, 37, 38). The physiopathology associated with lung damage during the acute phase includes infiltration of innate immune cells, cytokine storm, fibrosis, and thrombosis (39–43). However, it is unknown if these pathways also define long-term pulmonary sequelae after COVID-19. In this study, 60 subjects who had mild, moderate, or severe COVID-19 were evaluated according to the results of their CT scan and diffusing capacity of the lungs for carbon monoxide adjusted for hemoglobin (DLCOc) exam at 4-months post-infection, to identify patients with long-term pulmonary dysfunction (L-TPD). Once L-TPD was confirmed, we identified the main parameters supporting this sustained condition during the acute phase and 4-months after infection, and the concomitant long-term consequences at 12-months post-COVID-19.

## 2. Materials and methods

### 2.1. Study design

An observational and prospective cohort study was conducted following current recommendations from the STROBE statement (44). The study protocol was approved by the Institutional Review Board (45) from Servicio Salud BioBio (IRB:CEC113), and Servicio Salud Concepción (IRB: CEC-SSC:20-07-26), Chile. All patients and healthy controls (HCs) signed informed consent before entering the study, and all methods were performed in accordance with the Helsinki Declaration and Good Clinical Practice. Both, patients with COVID-19 and HCs, were between 18 and 70 years old. COVID-19 patients were recruited from Victor Rios Ruiz’s Hospital and Guillermo Grant Benavente’s Hospital and COVID-19 diagnosis was confirmed between April to July 2020 by positive SARS-CoV-2 PCR or radiological image during the acute phase and by the presence of anti-SARS-CoV-2 (Nucleocapside and Spike proteins) IgG antibodies, 4-months after acute infection. Healthy individuals were recruited from the University of Concepción, between April 2020 and August 2021, and the absence of COVID-19 was confirmed with negative PCR (weekly performed) and negative presence of SARS-CoV-2 specific antibodies. All participants were not vaccinated during the acute phase, nor at 4-months post-COVID-19, however, both patients and HCs were vaccinated between the 9- and 12-month period post-infection. We excluded elderly patients (more than 70 years old) and patients who were lost to follow-up, transferred to another hospital or city after discharge, and in palliative care, persistent oxygen requirement or mechanical ventilation, decompensate chronic comorbidities, or who had a mental disability that prevented the completion of evaluations. We also excluded previous pulmonary disease achieved by the medical record and self-report. Finally, pregnant women during the acute phase or during the follow-up were also excluded.

### 2.2. Clinical data

**FIGURE 1:**
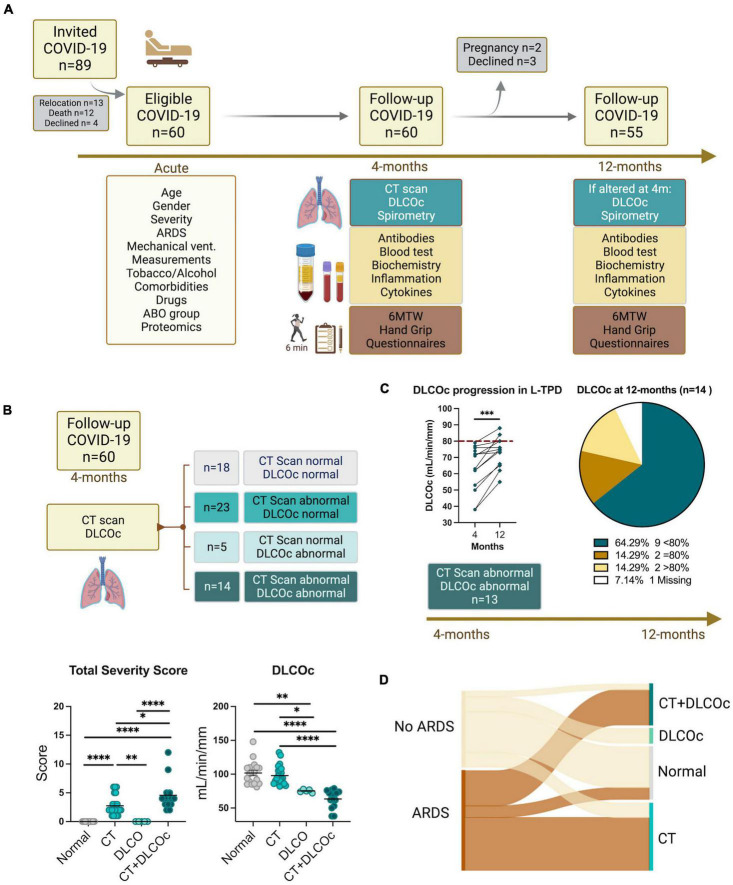
Study design flowchart. **(A)** A total of 89 patients with confirmed diagnosis of COVID-19 were invited to participate in the study, from which 29 were not included, resulting in a study cohort of 60 patients with different severity degree. Clinical and demographic data during acute phase and 4-months after COVID-19 was collected. **(B)** A computer tomography (CT) scan and diffusing capacity of the lungs for carbon monoxide (DLCO) exam were performed 4-months after acute COVID-19 defining abnormal CT scan total severity score (TSS) >1 and abnormal DLCO exam DLCOc <80%. Ordinary one-way ANOVA tests; ****p < 0.0001, ***p < 0.005, **p < 0.01, *p < 0.05. **(C)** The DLCO exam was reevaluated 12-months after acute infection in patients with abnormal CT scan and abnormal DLCO 4-months post infection. Before–after symbols and lines graph comparing the percentages of DLCOc in patients with L-TLD at 4- and 12-months post-infection; paired *t*-test ***p = 0.0003. Pie graph showing the percentage of patients with DLCOc <80%, DLCOc = 80%, DLCOc >80% and a missing value without follow-up due to pregnancy. **(D)** Sankey diagrams representing networks between COVID-19 severity during the acute phase and the level of pulmonary sequelae 4-month after infection according to the CT and DLCO exam.

To characterize pulmonary sequelae, 89 patients with COVID-19 were invited to participate in the study, from which 13 patients were relocated, 12 patients died, and 4 patients declined the invitation, resulting in a study cohort of 60 patients with different severity degrees (Figure 1A). Patients were recruited from Victor Rios Ruiz’s Hospital, Los Angeles and Guillermo Grant Benavente’s Hospital, Concepción, after informed consent, between March 2020 and June 2020. Patients were not vaccinated during the acute phase or 4-months after infection. However, the national vaccination program started between the 4- and the 12-month follow-up. COVID-19 patients were recruited and clinically evaluated by our medical team, reporting age, gender, ABO group, measurements (weight and height, neck, waist, and hip circumferences), body mass index [BMI, weight (Kg)/height (m)], tobacco history (current, former, or never smoker), alcohol usage (never, occasionally, and frequently), disease severity following the WHO recommendations (mild, moderate, and severe/critical), development of acute respiratory distress syndrome during the acute infection, symptoms, comorbidities at baseline [arterial hypertension, insulin resistance (IR), T2DM, heart failure, chronic obstructive pulmonary disease (COPD), cancer, chronic kidney disease (CKD), Atrial fibrillation, arrhythmia (Afib), coronary heart disease or stroke, congenital heart defects (CHD), non-alcoholic fatty liver disease (NAFLD) and hypothyroidism]. Sustained symptoms 4-months after infection and pulmonary tests were used to classify lung sequelae (Table 1).

**TABLE 1:** Clinical characteristic of the study cohort (*n* = 60).

	Normal *n* = 18	CT *n* = 23	DLCOc *n* = 5	CT + DLCOc *n* = 14	*p*-Value
**Gender**					
Male:female, *N* (%)	11:7 (61.1:38.9)	14:9 (60.9:39.1)	1:4 (20:80)	6:8 (42.9:57.1)	n.s.
Age (years), (SD)	35.6 ± 10.3	48.9 ± 10.3	44.8 ± 10.5	56.8 ± 11.9	<0.0001****
ABO group					n.s.
A, *N* (%)	3 (16.7)	5 (21.7)	2 (40)	4 (28.6)	n.s.
B, *N* (%)	2 (11.1)	1 (4.3)	1 (20)	2 (14.3)	n.s.
AB, *N* (%)	0 (0)	2 (8.7)	0 (0)	0 (0)	n.s.
O, *N* (%)	13 (72.2)	15 (65.2)	2 (40)	8 (57.1)	n.s.
**Measurements**					
Weight, Kg (SD)	85.1 ± 17.9	85.9 ± 15.3	78.7 ± 19.0	82.6 ± 11.5	n.s.
Height, m (SD)	1.68 ± 0.1	1.66 ± 0.1	1.59 ± 0.1	1.60 ± 0.1	n.s.
BMI, Kg/m^2^ (SD)	30.1 ± 5.1	30.9 ± 3.9	30.6 ± 4.9	32.8 ± 6.3	n.s.
Neck circumference, cm (SD)	41.4 ± 5.3	41.4 ± 4.5	41.2 ± 7.0	43.5 ± 5.9	n.s.
Waist circumference, cm (SD)	99.2 ± 14.3	105.0 ± 11.1	99.0 ± 11.9	108.8 ± 12.1	n.s.
Hip circumference, cm (SD)	105.1 ± 9.8	109.0 ± 8.5	108.2 ± 9.7	112.4 ± 9.8	n.s.
Tobacco status					n.s.
Current, *N* (%)	2 (11.1)	4 (17.4)	1 (20)	1 (7.1)	n.s.
Former, *N* (%)	3 (16.7)	6 (26.1)	2 (40)	4 (28.6)	n.s.
Never smoker, *N* (%)	13 (72.2)	13 (56.5)	2 (40)	9 (64.3)	n.s.
Alcohol usage					n.s.
Never, *N* (%)	7 (38.9)	8 (34.8)	3 (60)	7 (50)	n.s.
Occasionally, *N* (%)	11 (61.1)	15 (65.2)	2 (40)	5 (35.7)	n.s.
Frequently, *N* (%)	0 (0)	0 (0)	0 (0)	2 (14.3)	n.s.
COVID-19 severity					0.0013**
Mild, *N* (%)	11 (61.1)	3 (13.0)	3 (60)	1 (7.1)	0.0007***
Moderate, *N* (%)	4 (22.2)	5 (21.7)	2 (40)	6 (42.8)	n.s.
Severe/critical, *N* (%)	3 (16.7)	15 (65.2)	0 (0)	7 (50)	0.0031**
ARDS, *N* (%)	4 (22.2)	18 (78.3)	0 (0)	12 (85.7)	<0.0001****
**Symptoms during acute phase**					
Fever, *N* (%)	9 (50)	15 (65.2)	3 (60)	9 (64.3)	n.s.
Headache, *N* (%)	11 (61.1)	14 (60.9)	4 (80)	8 (57.1)	n.s.
Chest pain, *N* (%)	7 (38.9)	10 (43.5)	2 (40)	8 (57.1)	n.s.
Sore throat, *N* (%)	8 (44.4)	8 (34.8)	4 (80)	6 (42.9)	n.s.
Cough, *N* (%)	11 (61.1)	16 (69.6)	2 (40)	10 (71.4)	n.s.
Dyspnea, *N* (%)	11 (61.1)	18 (78.3)	1 (20)	14 (100)	n.s.
Polypnea, *N* (%)	8 (44.4)	16 (69.6)	1 (20)	11 (78.6)	0.045*
Myalgia, *N* (%)	15 (83.3)	13 (56.5)	4 (80)	7 (50)	n.s.
Desaturation, *N* (%)	1 (5.6)	2 (8.7)	0 (0)	0 (0)	n.s.
Abdominal pain, *N* (%)	9 (50)	6 (26.1)	1 (20)	3 (21.4)	n.s.
Diarrhea, *N* (%)	8 (44.4)	8 (34.8)	2 (40)	3 (21.4)	n.s.
Change smell, *N* (%)	7 (38.9)	10 (43.5)	3 (60)	5 (35.7)	n.s.
Change taste, *N* (%)	8 (44.4)	9 (39.1)	3 (60)	4 (28.6)	n.s.
**Comorbidities**					
Arterial hypertension, *N* (%)	4 (22.2)	7 (30.4)	0 (0)	9 (64.3)	0.020*
IR at baseline, *N* (%)	0 (0)	5 (21.7)	0 (0)	6 (42.9)	0.012*
T2DM at baseline, *N* (%)	1 (5.6)	4 (17.4)	0 (0)	3 (21.4)	n.s.
Heart failure, *N* (%)	0 (0)	0 (0)	0 (0)	0 (0)	
COPD, *N* (%)	0 (0)	0 (0)	0 (0)	0 (0)	
Previous cancer, *N* (%)	0 (0)	0 (0)	0 (0)	1 (7.1)	n.s.
CKD, *N* (%)	0 (0)	0 (0)	0 (0)	0 (0)	
Afib, *N* (%)	0 (0)	0 (0)	0 (0)	1 (7.1)	n.s.
Stroke, *N* (%)	0 (0)	0 (0)	0 (0)	1 (7.1)	n.s.
CHD, *N* (%)	0 (0)	0 (0)	0 (0)	0 (0)	
NAFLD, *N* (%)	2 (11.1)	1 (4.3)	1 (20)	3 (21.4)	n.s.
Hypothyroidism, *N* (%)	0 (0)	2 (8.7)	1 (20)	2 (14.3)	n.s.
**Therapy**					
ECA/ARA2, *n* (%)	0 (0)	5 (21.7)	0 (0)	7 (50)	0.0034**
Beta blockers, *n* (%)	0 (0)	0 (0)	0 (0)	3 (21.4)	0.0156*
Ca^++^ blq, *n* (%)	1 (5.6)	1 (4.3)	0 (0)	4 (28.6)	n.s.
Aldosterone inhibitor, *n* (%)	0 (0)	0 (0)	0 (0)	1 (7.1)	n.s.
Diuretic drugs, *n* (%)	0 (0)	1 (4.3)	0 (0)	3 (21.4)	n.s.
Metformin, *n* (%)	1 (5.6)	8 (34.8)	0 (0)	6 (42.9)	0.0313**
Insulin, *n* (%)	0 (0)	3 (13.0)	0 (0)	3 (21.4)	n.s.
Hyperlipemia drug, *n* (%)	2 (11.1)	4 (17.4)	1 (20)	5 (35.7)	n.s.
Combined ECA/ARA2 and metformin, *n* (%)	0 (0)	2 (8.7)	0 (0)	5 (35.7)	0.0112*
**4-months after COVID-19**					
Pulmonary test					
Abnormal CT, *N* (%)	0 (0)	23 (100)	0 (0)	14 (100)	<0.0001****
DLCO <80%, *N* (%)	0 (0)	0 (0)	5 (100)	14 (100)	<0.0001****
Symptoms					
Fever, *N* (%)	0 (0)	0 (0)	0 (0)	0 (0)	
Headache, *N* (%)	7 (38.9)	8 (34.8)	3 (60)	3 (21.4)	n.s.
Chest pain, *N* (%)	1 (5.6)	1 (4.3)	0 (0)	2 (14.3)	n.s.
Sore throat, *N* (%)	1 (5.6)	2 (8.7)	1 (20)	1 (7.1)	n.s.
Cough, *N* (%)	2 (11.1)	4 (17.4)	1 (20)	5 (35.7)	n.s.
Dyspnea, *N* (%)	1 (5.6)	7 (30.4)	1 (20)	6 (42.9)	n.s.
Polypnea, *N* (%)	0 (0)	2 (8.7)	1 (20)	1 (7.1)	n.s.
Myalgia, *N* (%)	1 (5.6)	3 (13.0)	0 (0)	3 (21.4)	n.s.
Desaturation, *N* (%)	0 (0)	0 (0)	0 (0)	0 (0)	
Abdominal pain, *N* (%)	1 (5.6)	0 (0)	0 (0)	0 (0)	n.s.
Diarrhea, *N* (%)	0 (0)	0 (0)	0 (0)	0 (0)	
Change smell, *N* (%)	2 (11.1)	1 (4.3)	0 (0)	1 (7.1)	n.s.
Change taste, *N* (%)	1 (5.6)	0 (0)	0 (0)	0 (0)	n.s.

BMI, body mass index; ARDS, acute respiratory distress syndrome; IR, insulin resistance; T2DM, type 2 diabetes mellitus; COPD, chronic obstructive pulmonary disease; CKD, chronic kidney disease; Afib, atrial fibrillation; CHD, coronary heart disease; NAFLD, non-alcoholic fatty liver disease. ACE, angiotensin-converting enzyme; *N*, number of patients; %, percentage; SD, standard deviation. Chi-square test; ****p < 0.0001, ***p < 0.0002, **p < 0.0021, and *p < 0.0332.

### 2.3. Pulmonary function test

Pulmonary function tests were assessed as previously reported by our research group (46). Briefly, first, an arterial blood sample was obtained for arterial blood gas analysis in the morning after an overnight fast. Then, all participants underwent forced spirometry at baseline and 15 min after inhalation of 400 μg of salbutamol (CPF−S/D; Medical Graphics Inc., USA). The procedure followed the current guidelines of the American Thoracic Society (ATS). Data from the forced vital capacity (FVC, %), forced expiratory volume in the first second (FEV1, %), FEV1/FVC ratio, and the maximum forced expiratory flow (FEFmax) were recorded. The diffusing capacity of the lungs for carbon monoxide (DLCO) and a six−minute walk test (6MWT) was performed. DLCO (Elite PlatinumDL; Medical Graphics Inc., USA) was corrected using the barometric pressure: hemoglobin (DLCOc), % ml/min/mm Hg, DLCOc 80%, alveolar volume (AV, %), and DLCO/AV ratio (%). DLCOc <80% was considered abnormal. For CT scan, all images were acquired using a high−resolution CT scan (SOMATOM, Siemens, Germany). The images and the classification (normal or abnormal chest CT) were defined by a radiologist blinded to the medical records, reporting: ground−glass opacities, mixed ground−glass opacities, consolidation, interlobular thickening, bronchiectasis, atelectasis, solid nodules, non-solid nodules, reticular lesions, fibrotic lesions, air trapping, and the number of lobes affected were considered. The total severity score (TSS) was used to quantify the abnormalities on chest CT, according to the visual inspection of each lobe, reporting the % impairment of each lobe (0–25%: 1 point; 26–50%: 2 points, 51–75%: 3 points, and 76–100%: 4 points), and the sum of each lobe represents the TSS. TSS >1 was considered abnormal CT. Test interpretation: Spirometry tests were analyzed between groups by measuring the FVC (the largest volume of air the patient forcefully exhales after deeply breathing), the forced expiratory volume (FEV_1_; the largest volume of air the patient forcefully exhales in one second), the FEV_1_/FVC ratio, and the FEFmax (the peak expiratory flow rate during expiration), all pre- and post-treatment with a bronchodilator.

### 2.4. The six-minute walk test

The 6MWT was performed in a 30-m-long corridor, indicating the start and end point through a plastic cone. Additionally, marks were made every 3 m (adhesive tape) to facilitate the evaluator’s measurement. Regarding the procedure, each patient had to remain at rest for 10 min prior to performing the test and then be evaluated through pulse oximetry and Borg scale at the beginning and at the end of the evaluation. Prior to the procedure, each patient received instructions for the preparation, objective, and instructions for the test based on the ATS Statement: Guidelines for the Six-Minute Walk Test (47).

### 2.5. Handgrip

A hydraulic hand dynamometer 200 lb/90 kgf baseline (Baseline^®^) was used to measure hand grip strength. This evaluation was performed with the subject seated in a chair with a backrest, shoulders adducted and without rotation, elbow flexed at 90°, forearm and wrist in a neutral position, feet flat on the floor with back supported. The dynamometer is positioned vertically and without limb support. The procedure consisted of performing a maximum grip force for 3 s, with a 1-min rest between each repetition, making two attempts (48). The Borg scale, which is the most used in the world of work, assigns an effort value between 1 and 10. If the force used in the task is “very, very weak” or almost absent, it is assigned the value of 0.5. On the contrary, if the required force is the maximum, the value 10 is assigned, for the procedure. For this research, a visual scale of 11 inches high was used (47, 49).

### 2.6. Questionnaire for physical and mental evaluation

In both visits, questionnaires that assess post-COVID-19 quality of life were included, such as the physical and mental short-form 12 questionnaire and the Hospital Anxiety and Depression Scale (HADS) questionnaire. Depression was assessed using the Beck Depression Questionnaire. Dyspnea was assessed by the modified Medical Research Council (mMRC). Muscle fatigue was measured by Chalder’s binary fatigue questionnaire. Participants’ sleep quality was assessed by different questionnaires targeting different parameters such as human circadian rhythms assessed by the Morningness-Eveningness Questionnaire (MEQ); Sleep health assessed by the Satisfaction, Alertness, Timing, Efficiency, and Duration (SATED) questionnaire and by the Pittsburg questionnaire; the sleepiness of the patients was measured by the Epworth Sleepiness Scale (ESS) accompanied by the evaluation of sleep apnea by means of the snoring, tiredness, observed apnea, blood pressure, BMI, age, neck circumference and sex (STOP-BANG) test and insomnia by the insomnia severity index (ISI). The personal change in the quality of life (QoL) of the participants after overcoming the SARS-CoV-2 infection was also evaluated using a visual analog scale with a range from 0% (worst QoL) to 100% (best QoL).

### 2.7. Laboratory data

Venous blood samples were collected with anticoagulant for hemogram and plasma collection and without anticoagulant for clinical biochemistry exams and serum collection from COVID-19 patients and HCs. The samples were obtained in the morning after an overnight fast. We evaluated the following laboratory parameters: (1) plasma glucose using a glucose-oxidase method, and total plasma cholesterol, HDL cholesterol, and triglycerides were assessed with standard enzymatic spectrophotometric technique. Plasma LDL was calculated by the Friedewald equation. Plasma insulin was measured using a radioimmunoassay. Homeostasis model assessment-estimated insulin resistance (HOMA-IR) was calculated according to: Fasting plasma glucose (mmol/L) times fasting serum insulin (mU/L) divided by 22.5. Total bilirubin, direct bilirubin, indirect bilirubin, albumin, globulin proteins, the albumin/globulin (A/G) ratio, hepatic enzymes alanine aminotransferase, aspartate aminotransferase (AST), gamma glutamyl transferase (GGT), and alkaline phosphatase (AP), LDH, phosphorus, calcium, uric acid, and cretinemia were determined by clinical biochemistry analysis using Biossays 240 Plus (Molecular Diagnostics). Hemogram was performed in Dymind 25 (Dymind DF 52).

### 2.8. Inflammatory parameters

Cytokines (IL-12, IL-1β, IL-6, IL-8, and TNF-α), chemokines (CCL5, CCL2, CXCL9, and CXCL10), and anaphylatoxins (C3a, C4a, and C5a) were measured with BD cytometric bead array (CBA) Human Inflammatory Cytokines Kit (Catalog No. 551811, BD), BD CBA Human Chemokine Kit (Catalog No. 552990, BD), and BD CBA Human Anaphylatoxin Kit (Catalog No. 561418, BD), respectively. All kits were acquired with LSR-Fortessa X20 (BD) and analyzed with FCAP Array Software v3.0 (BD Biosciences). Antibodies were measured with MAGLUMI 2019-nCoV IgM Kit (SNIBE) and MAGLUMI 2019-nCoV IgG Kit (SNIBE) (≥1.00 AU/ml) in a MAGLUMI 800 (SNIBE).

### 2.9. Artificial intelligence

Pulmonary variables such as DLCOc, spirometry test, questionnaires, 6MWT, demographic information, comorbidities, and measurements (height/weight) were tabulated and analyzed with machine learning, to identify the most relevant characteristics between the four groups (Supplementary Table 1). Random forest and XGBoost algorithms were applied to classify each group class (normal, DLCOc, CT, and CT + DLCOc) (Supplementary Table 2). The confusion matrix of the random forest classifier (Supplementary Figure 1A) resulted in a global accuracy of 93%, precision and recall ranged between 0.8 and 1.0, and F1-score ranged between 0.75 and 1.0. Some misclassification occurred in the “CT” class. With the XGBoost classifier, the confusion matrix (Supplementary Figure 1B) resulted in a global accuracy of 96%, precision ranged between 0.89 and 1.0, recall ranged between 0.67 and 1.0, and F1-score ranged between 0.80 and 1.0. A misclassification occurred in the “CT + DLCO” class. Both classifiers reveled very high AUC values in all groups (Supplementary Figures 1C, D). Then, the data was presented with a SHAP (SHapley Additive exPlanations) plot that represents the feature importance and the contribution of input variables to the XGBoost integration.

### 2.10. Machine learning

Patient and HC data previously described was tabulated and filtered to perform artificial intelligence (AI) analysis. All calculations were performed in Python 3.9. An unbalanced class distribution was observed in our data, thus imbalance class in the datasets was reduced with the SMOTE algorithm (50) from the imbalanced-learn library as an over-sampling method. This algorithm increases the sensitivity of a classifier to the minority class. Machine learning-based patient classification was performed by using the Scikit-learn library (51), the Random Forest Classifier, and the XGBoost algorithms. These ensemble methods combine predictions through estimators. The hyperparameter search was performed using GridsearchCV. The data were split into training data (80%) and test data (20%). The analysis of feature importance per group was examined using the Shapley Additive explanation algorithm (52) where the variables are ranked in descending order.

### 2.11. Proteomic methods

#### 2.11.1. Serum protein depletion

The serum proteins were depleted with HU-14 Protein Depletion Spin Columns (Agilent, USA), 800 μg of serum native proteins were added per column and the protocol suggested by the manufacturer was followed.

#### 2.11.2. Protein extraction and digestion for nLC-MS/MS

The previously depleted proteins were subjected to precipitation using 5:1 v/v cold acetone 100% v/v and incubated overnight at −20°C, then they were centrifuged at 15,000 × *g* for 10 min, the supernatant was discarded and the pellet was washed three times with acetone at 90% v/v, later the proteins were dried in a rotary concentrator at 4°C, and finally they were resuspended in 8 M urea with 25 mM of ammonium bicarbonate pH 8.0.

The proteins were reduced using a final concentration of 20 mM DTT for one hour, then they were alkylated incubating for 1 h with 20 mM iodoacetamide in the dark, then the proteins were quantified using the Qubit protein quantification kit and 10 μg of proteins. The total was diluted to 1 M urea using 25 mM ammonium bicarbonate pH 8.0, then the proteins were digested with trypsin/LyC (Promega) in a 1:50 ratio overnight at 37°C. The peptides were cleaned using SepPack Vac C18 (Waters, USA) using the protocol suggested by the manufacturer, the eluted peptides were dried using a rotary concentrator at 4°C and resuspended in 2% ACN with 0.1% v/v formic acid (MERCK, Germany), and quantified using Direct detect (MERCK Millipore).

#### 2.11.3. Peptide fractionation and library construction

High pH reversed-phase fractionation was performed on an ÄKTA Avant25 (General Electric) coupled to a refrigerated fraction collection. Purified peptides were separated on a reversed-phase column BHE 2.1 cm × 5 cm (Waters) at a flow rate of 0.2 ml/min at pH 10. The binary gradient started from 3% buffer B (90% ACN in 5 mM ammonium formate pH 10), followed by linear increases to the first 40% B within 30 min, to 60% B within 15 min, and finally to 85% B within 5 min. Each sample was fractionated into 24 fractions in 400 μl volume intervals. The fractions were dried in a vacuum-centrifuge and reconstituted in water with 2% ACN and 0.1% formic acid and concatenated in eight fractions.

Each fraction was injected into a nanoELUTE nano liquid chromatography system (Bruker Daltonics), peptides (200 ng of digest) were separated within 60 min at a flow rate of 400 nl/min on a reversed-phase column Aurora Series CSI (25 cm × 75 μm i.d. C18 1.6 μm) (IonOpticks, Australia) with 50°C. Mobile phases A and B were water and acetonitrile with 0.1 vol% formic acid, respectively. The %B was linearly increased from 2 to 17% within 37 min, followed by an increase to 25% B within 15 min and further to 35% within 8 min, followed by a washing step at 85% B and re-equilibration.

#### 2.11.4. The timsTOF Pro mass spectrometer

All fractions’ samples were analyzed on a hybrid trapped ion mobility spectrometry (TIMS) quadrupole time-of-flight mass spectrometer (TIMS-TOF Pro, Bruker Daltonics) via a CaptiveSpray nano-electrospray ion source. The MS was operated in data-dependent mode for the ion mobility-enhanced spectral library generation. We set the accumulation and ramp time was 100 ms each and recorded mass spectra in the range from m/z 100 to 1,700 in positive electrospray mode. The ion mobility was scanned from 0.6 to 1.6 Vs/cm^2^. The overall acquisition cycle of 1.16 s comprised one full TIMS-MS scan and 10 parallel accumulation-serial fragmentation (PASEF) MS/MS scans.

When performing DIA, we define quadrupole isolation windows as a function of the TIMS scan time to achieve seamless and synchronous ramps for all applied voltages. We defined up to 16 windows for single 100 ms TIMS scans according to the m/z-ion mobility plane. During PASEF MSMS scanning, the collision energy was ramped linearly as a function of the mobility from 59 eV at 1/K0 = 1.6 Vs/cm^2^ to 20 eV at 1/K0 = 0.6 Vs/cm^2^. Generation of spectral library and DIA-PASEF processing.

#### 2.11.5. Database searching and spectral library

Spectral library generation in FragPipe We used FragPipe computational platform (version 15) with MSFragger (version 3.2) (53, 54), Philosopher (version 3.4.13) (55), and EasyPQP^1^ (0.1.9) components to build spectral libraries. Peptide identification from tandem mass spectra (MS/MS) was done using the MSFragger search engine, using either raw (.d) files as input. Protein sequence databases *Homo sapiens* (UP000005640) from UniProt (reviewed sequences only; downloaded on 15 February 2021) and common contaminant proteins, containing in total 20,421 (*H. sapiens*) sequences were used. Reversed protein sequences were appended to the original databases as decoys. For the MSFragger analysis, both precursor and (initial) fragment mass tolerances were set to 20 ppm. Enzyme specificity was set to “stricttrypsin,” and either fully enzymatic peptides were allowed. Up to two missed trypsin cleavages were allowed. Oxidation of methionine, acetylation of protein N-termini, −18.0106 Da on N-terminal Glutamic acid, and −17.0265 Da on N-terminal Glutamine and Cysteine were set as variable modifications. Carbamidomethylation of Cysteine was set as a fixed modification. The maximum number of variable modifications per peptide was set to 3. The final spectral library was filtered to 1% protein and 1% peptide-level FDR.

DIA-NN configuration and dia-PASEF data processing DIA-NN 1.7.15 was used for the benchmarks and was operated with maximum mass accuracy tolerances set to a default average of 13 ppm for both MS1 and MS2 spectra. The two-proteome human was analyzed with match-between-runs enabled, Quantification mode was set to “Any LC (high accuracy).” All other settings were left default. DIA-NN’s output was filtered at precursor *q*-value <1% and global protein *q*-value <1%.

#### 2.11.6. Bioinformatic analyses

The quantification output reports from DIA-NN were exported and processed in the R statistical environment (56). The intensity values for each run are normalized by adjusting the medians. Missing values are imputed for each condition using the missForest algorithm (57). Significant differential expression of proteins was determined through a Bayes-based *t*-test (58). Any associated protein with a *p*-value < 0.05 is considered significant. The exploratory analysis like dimensional reduction and visualization of data were created using R v.3.6.0 with EnhancedVolcano (59), ComplexHeatmap v.2.0.0, (60) Rtsne (61), and base packages. The proteomic dataset including UniProt identifiers and logFC values of identified proteins in Mass spectrometry was submitted to ingenuity pathway analysis (IPA). Data were analyzed using IPA (QIAGEN Inc.).^2^ Core analysis was performed with the following settings: (i) indirect and direct relationships between molecules, (ii) based on experimentally observed data, and (iii) all data sources were admitted from the Ingenuity Knowledge Base.

### 2.12. Analysis of parameters in patients with heart infarction

Patients with cardiac infarction (Ethics Committee, reference number 15/LO/1998) gave written informed consent in accordance with the Declaration of Helsinki at Hospital Guillermo Grant Benavente.

A peripheral venous and a coronary blood sample were collected in tubes with EDTA from patients during surgery by the cardiologists of our team to obtain plasma and coronary or peripheral blood mononuclear cells (PBMCs). Blood was diluted with PBS 1:1 and PBMCs were obtained after centrifugation with Lymphoprep at 2,000 RPM for 20 min. Chemokines (IL-8, CCL5, CCL2, CXCL9, and CXCL10) were measured with BD CBA Human Chemokine Kit (Catalog No. 552990, BD) in the plasma and 1 × 10^6^ isolated PBMCs cells were stained with CD14, CD16, CD86, and Lox-1 (All from Biolegend) for 30 min at 4°C. Cells were acquired in an LSR-Fortessa X20 (BD) and analyzed with FlowJo (BD).

### 2.13. CXCR3 induction

CD14^+^ monocytes were isolated with Miltenyi Biotec kit (130-050-201) from PBMCs obtained from three healthy individuals. A total of 2 × 10^5^ monocytes were incubated with plasma from peripheral venous and coronary blood samples (1:4, plasma:media) from patients suffering cardiac infarction for 3 days at 37°C. Then, monocytes were stained with CD14 and CXCR3 (All from Biolegend) for 30 min at 4°C. Cells were acquired in an LSR-Fortessa X20 (BD) and analyzed with FlowJo (BD).

### 2.14. Migration assay

Monocyte chemotaxis was assessed using a 5-μm-pore Transwell filter system. CD14^+^ monocytes were isolated with Miltenyi Biotec kit (130-050-201) from PBMCs obtained from three healthy individuals. A total of 1 × 10^5^ monocytes were placed in the top chamber. The bottom chambers were filled with media, plasma from coronary samples from patients with cardiac infarction, and plasma from HCs (1:4, plasma:media). After 1 h at 37°C, cells were harvested from bottom compartments, counted using CountBright Absolute Counting Beads, and analyzed by flow cytometry. The percentage of migration for each subset was calculated as (number of monocytes in the bottom chamber after 60 min × 100)/initial number of monocytes in the top chamber.

### 2.15. CD16 phenotype

Peripheral blood mononuclear cells were obtained from COVID-19 patients after Ficoll density gradient centrifugation (Lymphoprep-Axis Shield). A total of 1 × 10^5^ PBMCs were stained immediately after isolation with CD14, CD16, CD86, MHC-II (All BioLegend) in two panels for 30 min at 4°C. Cells were acquired in an LSR-Fortessa X20 (BD) and analyzed with FlowJo (BD).

### 2.16. Statistical analysis

Statistical tests for clinical data were performed using Prism 9 Version 9.4.1 (458), software (GraphPad). Data are expressed as mean ± SD using individual values. Paired *t*-test were used to compare one variable between paired samples (DLCOs 4- vs. 12-months). Two-way ANOVA was used to compare BMI between 4 and 12 months from the same patient. Ordinary one-way ANOVA was used to compare clinical variables between patients’ groups. *Post-hoc* tests were used as indicated in the figure legends. *p*-Values are reported as follows: *p < 0.05, **p < 0.01, ***p < 0.001, and ****p < 0.0001.

## 3. Results

### 3.1. Structural and functional pulmonary sequelae characterization at 4- and 12-months post-COVID-19

To characterize pulmonary sequelae, 89 patients with COVID-19 were invited to participate in the study, from which 13 patients were relocated, 12 patients unfortunately passed away, and 4 patients declined the invitation, resulting in a study cohort of 60 patients with different severity degree (Figure 1A). Clinical and demographic data from our patient cohort during the acute phase was collected to report ARDS development, non-invasive or invasive mechanic ventilation, pharmacological therapy due to COVID-19, age, sex, comorbidities, and previous history of lung disease (Figure 1A). Our cohort included patients with severe, moderate, and mild COVID-19 according to the WHO recommendation (46, 62). Then, 4-months after acute COVID-19, patients were evaluated by measuring clinical biochemistry and inflammatory parameters, ABO group determination, SARS-CoV-2-specific IgM/IgG levels, medical exams, and functional tests. In addition, CT scans and DLCOc exams were performed to characterize lung dysfunction, and patients with abnormal CT scan [defined as the total severity score (TSS) >1] and abnormal DLCOc exam adjusted by hemoglobin (defined as DLCOc <80%) were identified as patients with L-TPD (Figure 1B). Our analysis revealed that 30.0% of patients had normal lung function, 38.3% of patients had abnormal CT scan only, 8.3% of patients had abnormal DLCOc exam only, and 23.3% patients had L-TPD (Figure 1B and Table 1). Despite patients with L-TPD had higher TSS scores than patients with abnormal CT scan only, suggesting a higher degree of pulmonary damage after COVID-19, the CT scan results alone were not resolutive to define L-TPD, thus the combination with the DLCOc exam confirmed both structural and functional lung dysfunction in patients with L-TPD. Since it was not clear whether lung dysfunction was reversible, the DLCOc was reevaluated in 13 of the 14 patients with L-TPD, at 12-months post-acute infection (1 excluded due to pregnancy), and we observed that despite the improvement of DLCOc percentages over time, more than 50% of the patients maintained DLCOc <80% a year after infection (Figure 1C), suggesting that longer evaluations are required to define the duration of this impairment. The demographic and clinical data from the patients revealed that age and only two comorbidities (hypertension and insulin resistance) were significantly associated with L-TPD (Table 1). In terms of severity, the data showed that patients with abnormal CT scan only and patients with L-TPD had higher frequencies of ARDS (Figure 1D) during the acute phase, suggesting that severity favored L-TPD, but was not a sole causal factor (Table 1). In fact, the CT and the CT + DLCOc patient groups were very similar during the acute phase of the disease, thus it was not clear why some patients were not able to recover lung function. In summary, our analysis defined L-TPD as patients with abnormal CT scan and DLCOc exam 4-months after infection, a state favored by age, ARDS development, and the presence of comorbidities such as hypertension and insulin resistance.

### 3.2. L-TPD was associated with reduced aerobic capacity and handgrip strength

**FIGURE 2:**
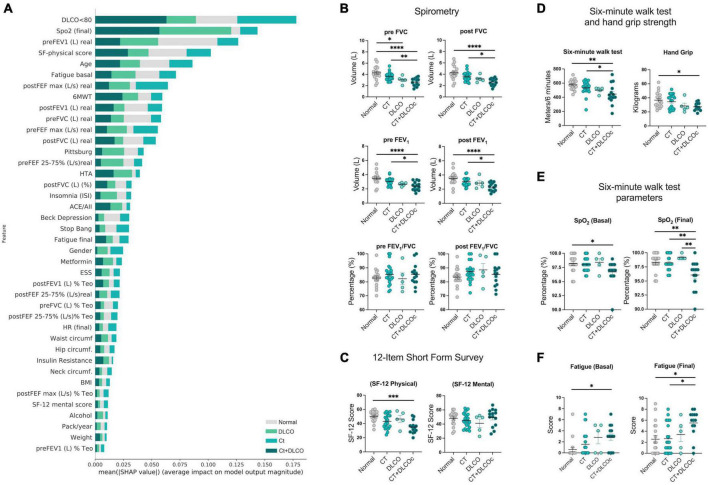
Reduced aerobic capacity and handgrip strength in L-TPD. **(A)** Shapley Additive exPlanations (SHAP) graph showing the contribution of functional features in the definition of lung sequelae according to a SHAP-value assigned by the algorithm. **(B)** Scatter plots of spirometry tests forced vital capacity (FVC), forced expiratory volume (FEV_1_), and FEV_1_/FVC ratio were compared between patient groups pre and post treatment with bronchodilator salbutamol. **(C)** Scatter plots of physical and mental 12-item short form survey scores between patient groups. **(D)** Scatter plots of distance walked in 6 min and hand grip test between patient groups. For the 6MWT, **(E)** oxygen saturation and **(F)** fatigue scores were measured before and after the test and compared between patient groups. For panels **(B–F)**, ordinary one-way ANOVA tests; ****p < 0.0001, ***p < 0.005, **p < 0.01, *p < 0.05.

To identify specific characteristics associated with sustained L-TPD, AI algorithms were used to determine the main variables (spirometry test, questionnaires, 6MWT, demographic information, and comorbidities) supporting L-TPD, 4-months post-acute infection (Matrix at Supplementary Figure 1 and Supplementary Table 2). Our SHAP plot showed that variables such as the spirometry exam, 6MWT, and the short form (SF)-physical questionnaire were the top features according to the mean SHAP value (Figure 2A). Thus, we analyzed these features to evaluate whether these parameters were impaired in L-TPD in comparison with other patient groups. Spirometry tests were analyzed between groups and the data revealed that patients with L-TPD had reduced FVC and forced expiratory volume (FEV_1_) in comparison with patients with no lung sequelae or patients with solely CT alteration, whereas no differences were observed regarding the FEV_1_/FVC ratio (Figure 2B), suggesting that patients with L-TPD have a restrictive lung condition. Since this condition impairs the lungs from fully expanding, limiting the volume of air and amount of oxygen that a person breathes in, it could favor fatigue and depression. Thus, we performed the physical and mental SF-12 questionnaire. The mental SF-12 questionnaire scores revealed no difference between groups, however in the physical SF-12 questionnaire, lower scores were obtained by patients with L-TPD (Figure 2C), thus we performed 6MWT and handgrip tests. The 6MWT was used as a validated measure of exercise capacity for patients, in which oxygen desaturation and fatigue scores were recorded before and after the test, whereas the handgrip test was used to measure the maximum isometric strength of the hand and forearm muscles. The results from the 6MWT demonstrated that patients with L-TPD walked fewer meters than the control group and the CT-group (Figure 2D), and had less handgrip strength than the control group (Figure 2D). Before the 6MWT, patients with L-TPD had lower oxygen desaturation values (Figure 2E) and higher fatigue scores (Figure 2F) in comparison with the control group, however, after the 6MWT (Final), this difference was also observed between patients with L-TPD and the CT-group. In summary, we demonstrated that patients with L-TPD exhibited a restrictive lung condition, as well as, reduced aerobic capacity and reduced muscular strength.

### 3.3. Circulating chemokine CXCL9 and platelet counts are augmented in patients with L-TPD post-COVID-19

**FIGURE 3:**
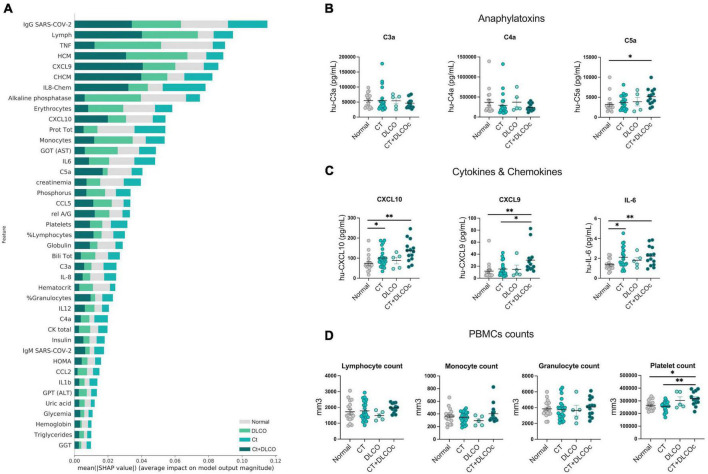
Inflammatory parameters sustained in L-TPD. **(A)** Shapley Additive exPlanations (SHAP) graph showing the contribution of circulating features in the definition of lung sequelae according to a SHAP-value assigned by the algorithm. **(B)** Scatter plots of anaphylatoxins C3a, C4a, and C5a between patient groups. **(C)** Scatter plots of CXCL10, CXCL9, and IL-6 levels between patient groups. **(D)** Scatter plots of lymphocyte, monocyte, granulocyte, and platelet cell counts between patient groups. For panels **(B–D)**, ordinary one-way ANOVA tests; **p < 0.01 and *p < 0.05.

Since intrinsic restrictive lung diseases usually result from inflammation and scarring of lung tissue, we evaluated systemic factors to identify specific variables that may support lung-dysfunction. Using a similar approach to Figure 2A, systemic variables such as blood tests, clinical biochemistry parameters, insulin, inflammatory parameters (cytokines, chemokines, and anaphylatoxins), hemogram, and antibodies 4-months post-COVID-19 were tabulated and analyzed with machine learning algorithms. A SHAP plot showed that variables such as chemokines, cytokines, and anaphylatoxins were increased in the L-TPD group (Figure 3A). Thus, we analyzed anaphylatoxins and we observed a significant difference between patients with L-TPD and the control group for C5a levels, but not for C3a and C4a (Figure 3B). Several cytokines and chemokines were also analyzed, but we only found significant differences for CXCR3 ligands CXCL10, CXCL9, and IL-6. Whereas patients with abnormal CT and L-TPD showed higher levels of CXCL10 and IL-6 in comparison with the control group, CXCL9 levels were increased in the L-TPD group in comparison with the control and patients with abnormal CT scan only (Figure 3C). No significant differences were observed between groups for IL-12, IL-1β, IL-10, IL-8, TNF-α, CCL5, and CCL2. Analysis from blood tests showed no differences regarding lymphocyte, monocyte, and granulocyte cell counts (Figure 3D), however, patients with L-TPD exhibited a higher number of platelets in comparison with the control group and patients with abnormal CT scan (Figure 3D). Overall, our data showed that CXCL9 and platelet counts were the main circulating variables supporting L-TPD in comparison with patients without sequelae or with abnormal CT scan only, whereas C5a, CXCL10, and IL-6 also favored L-TPD, but their levels were not significantly different than the ones from the CT-group.

### 3.4. Patients with L-TPD after COVID-19 exhibited metabolic sequelae 12-months post-COVID-19

**FIGURE 4:**
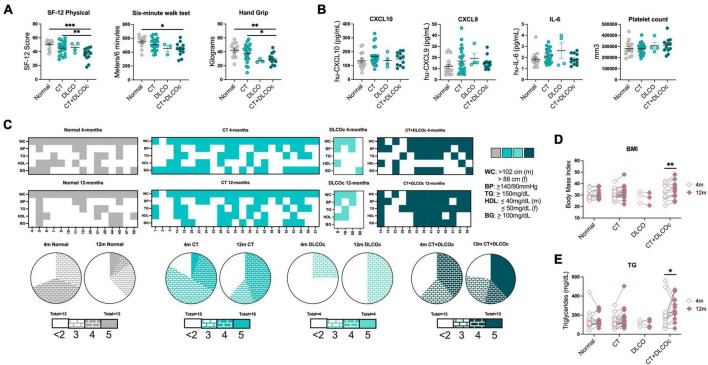
Metabolic syndrome in L-TPD a year post-COVID-19. **(A)** Scatter plots of SF-12 physical, distance walked in 6MWT and hand grip test between patient groups at 12-months post-COVID-19. **(B)** Scatter plots of CXCL10, CXCL9, and IL-6 levels and platelets counts between patient groups at 12-months post-COVID-19. **(C)** Heatmaps representing individual patients in the *x*-axes and metabolic syndrome parameters in the *y*-axes for the different patient groups at 4- and 12-months post infection. Colored squares represent that the patient exhibited. Waist circumference (WC >102 cm for male and WC >88 cm for female), blood pressure (BP ≥140/90 mmHg), triglycerides (TG ≥150 mg/dl), HDL-cholesterol (HDL ≤40 mg/dl for male and HDL ≤50 mg/dl for female), and fasting blood glucose (BG ≥100 mg/dl). Then, pie charts compare the distribution of patients that exhibit <2, 3, 4, and 5 altered metabolic syndrome parameters between 4- and 12-month post infection for the different groups. **(D)** Pair comparison of body mass index and **(E)** triglycerides in patient groups between 4- and 12-months post-COVID-19. For panels **(A,B)**, ordinary one-way ANOVA tests; ***p < 0.005, **p < 0.01, *p < 0.05. For panels **(D,E)**, two-way ANOVA with Sidak multiple comparison tests; **p < 0.01 and *p < 0.05.

After characterizing patients with L-TPD 4-months after COVID-19, we evaluated the consequences at 12-months post-infection in comparison with the responses at 4-months. Interestingly, the physical SF-12 questionnaire showed higher significant differences between L-TPD and the other groups (Figure 4A) in comparison with the analysis at 4-months (Figure 2C). In addition, L-TPD walked fewer meters than the control group, however, no significant difference was observed between L-TPD and the CT-group (Figure 4A). In terms of strength, patients with L-TPD maintained lower handgrip scores, and therefore maintained reduced muscular strength a year post-infection (Figure 4A). When inflammatory parameters were compared between groups, we observed that the differences reported at 4-month were no longer observed at 12-months for CXCL10, CXCL9, IL-6, and platelet counts (Figure 4B). Since the aerobic capacity and muscular strength were reduced in patients with L-TPD than in other groups, changes in metabolic syndrome parameters and changes in BMI at 1-year post-COVID-19 in comparison with 4-months were evaluated considering waist circumference (WC), blood pressure (BP), triglycerides (TG), HDL levels and fasting blood glucose (BG). Heatmaps showing individual parameters per patient and pie charts summarizing the patient group data revealed that the L-TPD was the patient group that worsened the presence of metabolic syndrome parameters (Figure 4C). Interestingly, these observations in patients with L-TPD were associated with an increment in BMI (Figure 4D) and triglycerides (Figure 4E) at 12-months in comparison with 4-months. The fact that patients with L-TPD transfer less oxygen from the lungs to blood and therefore to tissues suggests a state of sustained hypoxia (63), that could modify metabolic pathways in the L-TPD patients by affecting cellular metabolism and reducing overall physical activities (45). In summary, patients with L-TPD worsened metabolic syndrome a year after COVID-19, thus it is relevant to follow-up the metabolic parameters periodically after COVID-19, especially in patients who had severe disease, in order to prevent sequelae and perform adequate dietary and physical exercise intervention.

### 3.5. Cardiac dysfunction, CXCL9, and chemotaxis of phagocytes support long-term pulmonary dysfunction in long-COVID-19 patients

**FIGURE 5:**
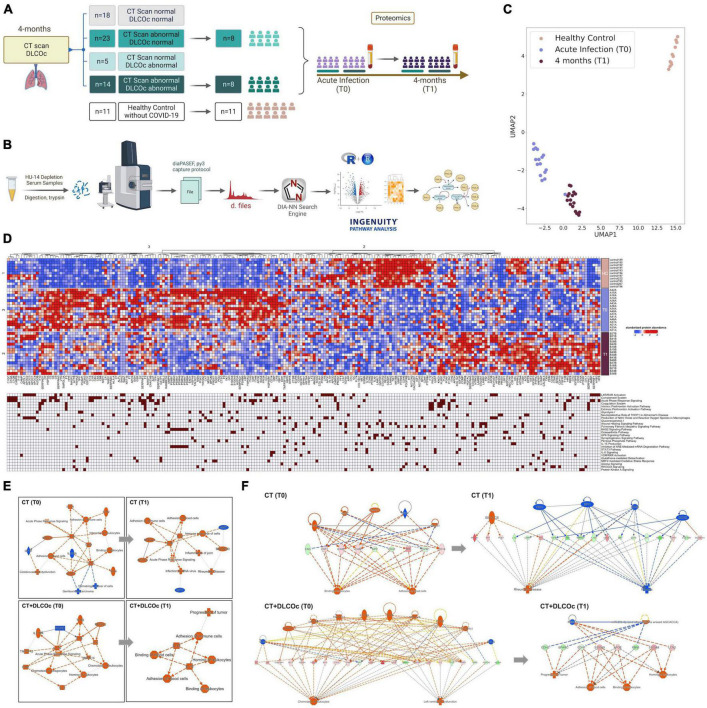
Cardiac dysfunction and chemotaxis are the main predicted annotations in L-TPD during acute COVID-19. **(A)** From the study cohort of 60 patients, 16 patients were selected from the CT (*n* = 8) and CT + DLCOc (*n* = 8) group and healthy controls (*n* = 11) without COVID-19. Serum from patients were collected during the acute phase and during the 4-month follow-up. **(B)** Serum samples were processed and acquired with TIMS-TOF Pro and the data was analyzed with R and IPA. **(C)** Principal component analysis of the protein profiling analyzed in samples that passed quality control, obtaining data from 11 healthy controls and the 16 patients during the acute (T0) and at 4 months post infection (T1) and **(D)** heatmap showing the proteins from serum differentially present between the different groups and their respective association with canonical pathways. **(E)** Overview of the main biological themes and **(F)** network regulators during the acute phase (T0) and 4-month follow-up (T1) after COVID-19 between patients who exhibited only CT scan abnormalities versus L-TPD (CT + DLCOc), considering canonical pathways, upstream regulators, diseases, and biological functions, showing a positive *z*-score in orange and a negative *z*-score in blue.

After identifying specific variables and physiological consequences associated with L-TPD, we finally evaluated the serum proteome profiles of a subset of patients from our cohort during the acute phase and 4-months after infection. Since the CT and the CT + DLCOc were very similar regarding the severity and clinical characteristics in the acute phase, but different in their evolution at 4- and 12-months after infection, we focused our attention on these two groups. We included 16 patients who developed ARDS during acute COVID-19, from which 8 patients developed L-TPD, and 8 patients only exhibited abnormal CT scan, 4-months after acute COVID-19 (Figure 5A and Supplementary Table 3). In addition, healthy individuals without COVID-19, confirmed with negative PCR (weekly performed for 4 months) and negative presence of SARS-CoV-2 specific antibodies before analysis, were included as controls (Figure 5A). The serum proteome was analyzed with mass spectrometry, obtained during the acute phase and in the 4-months follow-up (Figure 5B and Supplementary Figure 2). Samples from HCs, COVID-19 during the acute phase (T0), and COVID-19 at 4-months after infection (T1) were analyzed using Uniform Manifold Approximation and Projection (UMAP), a dimension reduction technique, showing the presence of three well-defined groups (Figure 5C). In addition, a heatmap revealed the differential presence of several proteins per group, which were associated with relevant pathways differentially activated between patients and HCs according to IPA (Figure 5D). Then, IPA analysis was used to determine significant disease or function annotations with predictive activation state in patients with L-TPD and patients with abnormal CT scan (Figure 5E). The graphical summary during the acute phase (T0) and at 4-month follow-up (T1) in Figure 5E provides an overview of the main biological themes and the relation between them. The data revealed that IFN-γ-mediated signaling was present in CT, whereas chemotaxis of phagocytes and leukocytes was present in patients with L-TPD, suggesting that patients with L-TPD did not promote an optimal IFN-γ-mediated response in the acute phase. Interestingly, when Th1 chemo-attractants CXCL10 and CXCL9 were evaluated during the acute phase, CXCL9 was increased in L-TPD versus patients with CT scan abnormalities (Supplementary Figure 3), suggesting that CXCL9 may be a compensating signal to recruit CXCR3-expressing cells, such as Th1 cells. Then, we analyzed networks with the corresponding upstream regulators, effector molecules, and downstream pathways (Figure 5F). In this case, chemotaxis of leukocytes and left ventricular dysfunction were upregulated pathways in L-TPD during the acute phase, whereas the progression of tumor, blood cell adhesion, and leukocyte binding were upregulated 4-months after disease. For patients with CT, binding and adhesion of blood cells were upregulated during the acute phase, whereas fibrosis was downregulated in the follow-up. Finally, when categories of tox functions and upstream regulators were analyzed in CT and CT + DLOCc subgroups, the data showed that cardiac dysfunction was the main pathway significantly activated in CT + DLOCc, suggesting that having cardiac dysfunction during the acute phase supports long-term pulmonary sequelae after COVID-19 (Supplementary Figure 4A). Moreover, unique upstream regulators (Supplementary Figure 4B) showed that IL-6 was relevant in the CT + DLOCc subgroup, whereas lipopolysaccharide, microtubule-associated protein tau (MAPT), and IFN-γ were relevant in the CT subgroup (Supplementary Figure 4C). All relevant disease or function annotations between the CT and CT + DLOCc subgroups, with the relevant proteins, are described in Supplementary Table 4. Overall, our data suggested that cardiovascular dysfunction and chemotaxis were the main pathways associated with the development of L-TPD.

### 3.6. CXCL9 is associated with heart dysfunction and migration of CD14^+^ phagocytes

**FIGURE 6:**
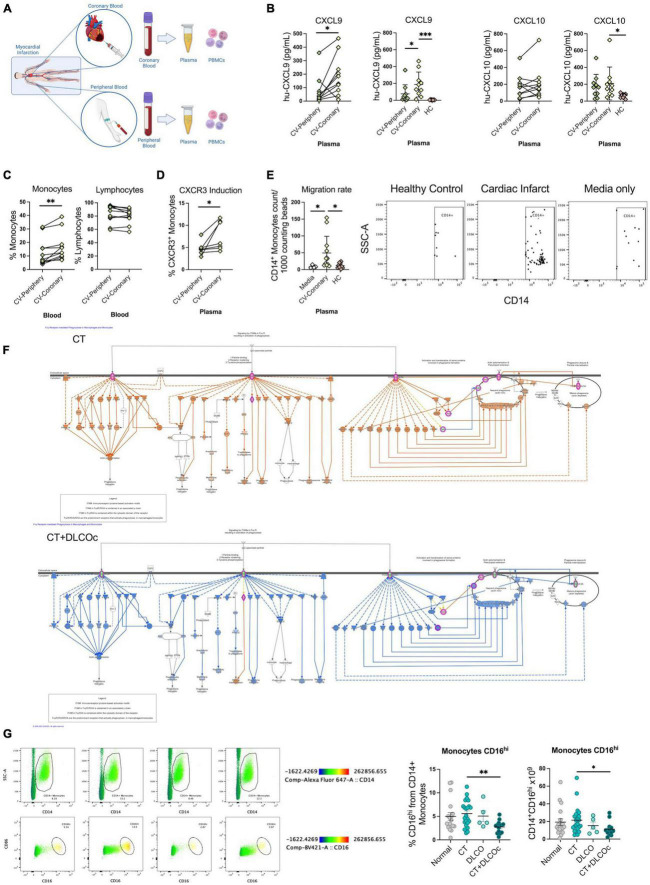
CXCL9 and monocyte chemotaxis are associated with myocardial infarction, however monocytes from L-TPD exhibited reduced expression and function of CD16. **(A)** Flowchart of coronary and peripheral blood samples obtained from patients suffering myocardial infarction. **(B)** Chemokine levels in plasma from coronary and peripheral blood samples. **(C)** Percentages of total CD14^+^ monocytes and lymphocyte (T, B, and NK) present in coronary and peripheral blood samples. **(D)** Percentage of CXCR3^+^ monocytes in the presence of plasma from coronary and peripheral blood samples for 72 h. **(E)** Representative dot plots and percentage of migrated monocytes to media, plasma from coronary and plasma from healthy control samples. The percentage of migration for each subset was calculated as (number of cells in the bottom chamber after 1 h × 100)/initial number of cells in the top chamber. **(F)** Ingenuity pathway analysis graphical representation of FCgamma receptor mediated phagocytosis in macrophages and monocytes in the CT (top) and CT + DLCO (bottom) groups at 4-months post infection. **(G)** Scatter plots and representative dot plots of CD16 expression in CD14^+^ monocytes from the different patient groups. For panels **(B–D)**, paired *t*-tests and for panels **(E,G)**, ordinary one-way ANOVA tests; ***p < 0.005, **p < 0.01, *p < 0.05.

In order to understand how chemotaxis and heart dysfunction may support L-TPD, we analyzed the presence of leukocytes and chemokines in patients suffering coronary infarction as a model of heart dysfunction. Thus, coronary, and peripheral blood samples from patients without COVID-19 were obtained during coronary infarction (Figure 6A and Supplementary Table 5). The data showed that CXCL9 (Figure 6B) and monocytes (Figure 6C) were the main chemokine and cell subset significantly augmented in coronary blood during coronary infarction in comparison with peripheral blood, indicating that CXCL9 is an inflammatory mediator of vascular damage. We then evaluated the induction of the CXCL9/10 receptor (CXCR3) in monocytes by plasma from patients with coronary infarction and CXCR3 was induced when healthy monocytes were co-cultured with plasma from patient samples (Figure 6D). Moreover, chemotaxis analysis demonstrated that plasma from patients with coronary infarction induced chemotaxis of CD14^+^ monocytes (Figure 6E), thus sustained sequelae could be supported by vascular inflammation mediated by increased levels of CXCL9 and monocyte migration due to heart dysfunction during acute COVID-19. Having shown the relevance of monocyte and chemotaxis in this context, we then analyzed monocyte/macrophage-related pathways from the proteomic data in both groups. Interestingly, we observed that whereas pathways from the CT group showed activated FCgamma receptor-mediated phagocytosis pathway, the same pathway was completely inhibited in the CT + DLCOc group (Figure 6F), suggesting that despite the persistent chemoattractant signal of monocytes, these were dysfunctional, mainly due to FCgamma receptor signaling. Other pathways in monocytes/macrophages such as the production of NO and ROS species in macrophages and the Liver X receptor/retinoid X receptor (LXR/RXR) Activation in monocytes showed no difference in patients with L-TPD in comparison with the CT group (Supplementary Figures 5, 6). Since the FCgamma receptor signaling pathway is triggered by FCGR1A/2A/3A, we analyzed the expression of FCGR3A (CD16) in monocytes from our COVID-19 patient cohort. The analysis of the percentage of CD16*^hi^*CD14^+^ cells from peripheral blood at 4-months post-infection revealed that patients with L-TPD exhibited lower percentages and numbers of CD16*^hi^*CD14^+^ monocytes than the CT group (Figure 6G), suggesting a reduced capacity to induce IgG-dependent cellular phagocytosis. Interestingly, this could be associated with the IFN-γ-mediated signaling observed during the acute phase, since this cytokine has been associated with CD16 induction in monocytes (64). Overall, our data suggest that mediators of cardiac dysfunction and chemotaxis of leukocytes in the context of SARS-CoV-2 infection contribute to alveolocapillary barrier damage during acute COVID-19, affecting the ability of the lungs to transfer oxygen to blood during the recovery phase, demonstrated by the reduced DLCOc percentages and the structural lung damage in patients with L-TPD. This persistent state of lung dysfunction and vascular inflammation promotes a restrictive lung condition in patients with L-TPD, which exhibits reduced aerobic capacity and reduced muscle strength. Furthermore, patients with L-TPD exhibited an inhibited FCgamma-receptor-mediated-phagocytosis pathway, suggesting an impair phagocytosis capacity of virus-antibody immune complexes. Finally, even though L-TPD patients improved lung function and inflammatory parameters between 4- and 12-months post-infection, this patient group increased the number of altered metabolic syndrome parameters and increased BMI, suggesting that metabolic sequelae is a further collateral consequence of L-TPD.

## 4. Discussion

In this study, we aimed to identify COVID-19 patients with long-term lung alterations and the main mediators associated with this persistent pulmonary dysfunctional state after COVID-19. We included a study cohort of 60 patients who had mild, moderate, or severe COVID-19 and we defined L-TPD as patients who had an abnormal CT scan and abnormal DLCOc exam 4-month post-infection. Our approach was to analyze all the measured variables (demographic, clinical, experimental, blood test, pulmonary function, function tests, and questionnaires) by using machine learning algorithms to identify the most relevant features between groups, to further analyze whether they were affected in the L-TPD group. Our main conclusions were that L-TPD was associated with advanced age, ARDS development, and the presence of hypertension and insulin resistance. In addition, during the acute phase, heart-related dysfunction and chemotaxis were also defining further development of L-TPD, suggesting that a phenomenon of immune-thrombosis was triggering pathways resulting in prolonged pulmonary dysfunction 4-months after infection. According to serum proteome analysis, this phenomenon was apparently supported by an impaired IFN-γ signaling-mediated pathway in the L-TPD. At 4-months, the L-TPD state was associated with a restrictive lung disease, according to the results from the spirometry showing lower lung capacity, resulting in reduced aerobic capacity, more fatigue, and reduced strength compared to other patient groups. In terms of inflammatory parameters, CXCL9 was the main systemic inflammatory parameter associated with L-TPD, whereas in terms of blood cell subsets, platelets were the only population significantly increased in L-TPD. In addition to those inflammatory factors, pathways associated with the progression of tumor, blood cell adhesion, and leukocyte homing were active at 4-months after disease in L-TPD, whereas FCgamma-mediated phagocytosis was inhibited in comparison with patients with CT scan altered, mainly due to reduced CD16 expression in L-TPD monocytes. Finally, 1-year post-infection, patients with L-TPD worsened metabolic syndrome and augmented BMI in comparison with other patient groups.

Long COVID-19 or post-COVID-19 has been recently proposed as a disease related to COVID-19-derived prolonged symptoms beyond 12 weeks after acute SARS-CoV-2 infection and not attributable to other possible causes (27). These manifestations are diverse according to all the organs that SARS-CoV-2 affects, for example, we have already described sleep health problems (65), erectile dysfunction (66, 67), and fatigue (46, 68). In terms of pulmonary sequelae, it has been described that it can include structural and functional damage (25, 37), which can be measured with CT (69), DLCO (70), and spirometry tests (28). In this context, it has been proposed that most of the patients who developed ARDS and required invasive mechanic ventilation, exhibited CT abnormalities 3–4 months post-COVID-19 (46, 68, 71), which improved over time (33, 72–74). Furthermore, it has been shown that the use of mechanic ventilation influences the lung structural alterations detected by the CT scan (72, 75–78). Therefore, is crucial to use another functional test to support these results. For this reason, spirometry and DLCO have been incorporated to analyze functional pulmonary dysfunction after COVID-19 (79, 80). In an initial approach, several combinations were evaluated to define L-TPD in our cohort (46), including TSS score, DLCOc >80%, spirometry (FVC >70%), however, exhibiting an abnormal CT scan in combination with a DLCOc >80% at 4-months after infection resulted as the best approach associated with functional impairment and inflammation in the post-COVID-19 patients. Our data suggest that early identification of patients with L-TPD requires a standardized evaluation of post-COVID-19 pulmonary sequelae in the clinic to apply appropriate interventions aimed at promoting full recovery and reducing pulmonary dysfunction. In addition, since abnormal DLCOc exams were still present in several patients with L-TPD 1 year after acute COVID-19, it is relevant to continue with the clinical monitoring of these patients beyond 12 months of infection to identify recovery periods regarding lung function or potential permanent tissue damage that will require lifelong therapy. In this regard, a study in the Chinese population has shown a reduction in the DLCOc from 1 to 2 years after COVID-19 (81), whereas a Spanish cohort has demonstrated a sustained improvement, not only in DLCOc, but also in the TSS 2 years after COVID-19 (82). It remains unknown the progression of lung dysfunction post-COVID in the Chilean population. It will be also relevant to monitor the long-term progression of metabolic syndrome and insulin resistance in the L-TPD group.

Cytokine storm and the exacerbated immune response have been associated with the development of ARDS in COVID-19 (83–85). IL-6 is the major regulator of acute phase protein synthesis in the liver (86), supporting the synthesis of C reactive protein, serum amyloid A, fibrinogen, and others. CXCL10 and CXCL9 are CXCR3 ligands that induce chemotaxis to the site of inflammation of several immune cells such as NKs, CXCR3^+^ T cells, and macrophages (87, 88). The complement system has been proposed as one of the most relevant pathways to define severe COVID-19 during the acute phase (40, 89, 90). The role of these inflammatory mediators has been described during the acute phase of COVID-19 and their changes are concordant with the anti-viral immune response, however, after infection, their levels return to normal conditions (78). Therefore, which pathways sustain the persistent presence of these inflammatory factors in L-TPD is still unclear. It has been proposed that failure in the viral clearance (91), a sustained hypoxic state (27, 92), and according to our data, cardiac dysfunction may promote prolonged damage, oxidative stress, endothelial dysfunction, sustained inflammation (93–95), and immuno-thrombosis (96, 97). Interestingly, the CD16-mediated phagocytosis pathway and the IFN-γ signaling-mediated network were impaired in L-TPD, suggesting that patients with L-TPD were chemoattracting immune cells and promoting inflammation, without developing an optimal anti-viral immune response.

The repercussions that occur in survivors after overcoming the COVID-19 disease are diverse and can normally extend between 4 and 12 weeks after the acute COVID-19, but there is a group of patients in whom these consequences are present beyond 12 weeks, without being attributable to any alternative diagnosis after acute COVID-19 (27). A persistent altered state of health after acute COVID-19 can be linked to greater age and severity during the pathology (98). According to several investigations, these prolonged post-COVID-19 consequences periodically lie in neurological problems such as depression, sleep disorders, and headaches, muscle fatigue, or weakness (25, 27, 70, 99), and sometimes respiratory problems that can persist even more than 12-months after acute COVID-19 (100). Also, it has been described that these consequences extend even further, leading to a post-COVID-19 multisystemic problem that is based on a chronic and prothrombotic inflammatory state, triggering hormonal imbalances by altering the correct function of the hypothalamic-pituitary-adrenal axis (101). This causes multiple metabolic disorders, which have already been identified among subsequent COVID-19 patients, for example, lipid disorders with a high load of LDL cholesterol, total cholesterol, and triglycerides, liver problems, a high concentration of glycosylated hemoglobin, diabetes mellitus, or obesity (102, 103). These metabolic consequences are more pronounced among patients who suffered symptomatic COVID-19 than those who presented asymptomatic disease, suggesting a greater heterogeneity of biochemical states in patients with persistent symptoms after COVID-19 (5). Thus, post-COVID-19 control of metabolic diseases such as diabetes mellitus or other comorbidities is extremely important, and intervention with physical exercise and adequate nutrition could also help to reduce the prolonged symptoms of COVID-19 (103). It has been widely described that a personalized and supervised follow-up of long-COVID patients with a multidisciplinary approach from diverse health partners, significantly reduces long-term lung sequelae (104). This treatment includes exercise-based therapy (personalized endurance, strength, and inspiratory muscle training). In addition, the literature reported that patient education, psychosocial counseling, diet control, and smoking cessation are fundamental aspects of the program to obtain a successful outcome (105–107).

We have described for the first time the inflammatory phenotype and the metabolic consequences of developing validated L-TPD after COVID-19, indicating the main factors to be considered 12-months after infection, such as metabolic syndrome and insulin resistance. Furthermore, we have demonstrated the association between lung dysfunction and sustained vascular inflammation, indicating that these patients require close follow-up to control the incidence of thrombosis. Finally, besides cardiovascular networks, our study revealed that the progression of tumor by miR-29b-3p was one of the top network regulators in L-TPD at 4-months post-COVID-19, thus, miR-29b-3p could become a potential biomarker of cell cycle dysregulation in patients with long-COVID.

The number of patients is the main limitation of this work, however, since mild, moderate, and severe patients were included, we were able to have a representative cohort according to disease severity. Proteomic analysis was performed only in a subset of severe patients and HCs because we did not have samples from all patients during the acute phase, especially the mild cases, due to the restrictions at the beginning of the pandemic. An international consensus about how to diagnose COVID-19-derived L-TPD has not been defined, therefore other evaluations may be required to improve our characterization. This study started in 2020, thus the information regarding sequelae was extremely limited at that time. In addition, the effect of vaccination was not studied in our cohort since vaccines were not available until the final evaluation, however, a recent study from the Mayo Clinic reported that getting a COVID-19 vaccination before viral infection, significantly reduced the symptoms of post-COVID conditions, promoting improved morbidity and function (108). All patients were quarantined during the study, thus besides the disease, external factors such as psychological stress, anxiety, and reduced mobility could also affect the outcome of the patients.

## Data availability statement

The datasets presented in this study can be found in online repositories. The names of the repository/repositories and accession number(s) can be found in the article/Supplementary material.

## Ethics statement

The studies involving humans were approved by the Servicio de Salud BioBio–Chile (IRB: CEC113) and Servicio de Salud Concepción–Chile (IRB: CEC-SSC:20-07-26). The studies were conducted in accordance with the local legislation and institutional requirements. The participants provided their written informed consent to participate in this study.

## Author contributions

SS: Methodology, Writing - original draft, Writing - review and editing. MV: Methodology, Writing - original draft, Writing - review and editing. MH: Methodology, Writing - review and editing. MH-B: Methodology, Writing - review and editing, Supervision. CC: Methodology, Writing - review and editing. RQ: Methodology, Writing - review and editing. BA: Methodology, Writing - review and editing. KA: Methodology, Writing - review and editing. DC: Methodology, Writing - review and editing. FL: Methodology, Writing - review and editing. MF: Methodology, Writing - review and editing. MN: Methodology, Writing - review and editing. EC: Methodology, Writing - review and editing. PL: Methodology, Writing - review and editing. AM: Methodology, Writing - review and editing. JL: Methodology, Writing - review and editing. JG: Methodology, Writing - review and editing. PG: Methodology, Writing - review and editing. BR: Methodology, Writing - review and editing. JB: Methodology, Writing - review and editing. RS: Methodology, Writing - review and editing. VO: Methodology, Writing - review and editing. PS: Methodology, Writing - review and editing. CV: Methodology, Writing - review and editing. GN: Methodology, Writing - review and editing. EK: Methodology, Writing - review and editing. FZ: Methodology, Writing - review and editing. LL: Methodology, Writing - review and editing. PB: Methodology, Writing - review and editing. EG-G: Methodology, Writing - review and editing. CT: Methodology, Writing - review and editing. LF: Methodology, Writing - review and editing. GC: Methodology, Writing - review and editing. UW: Funding acquisition, Writing - review and editing. ER: Funding acquisition, Writing - review and editing. M-IY: Funding acquisition, Writing - review and editing. BM: Funding acquisition, Writing - review and editing. GL: Funding acquisition, Writing - review and editing. DG-C: Funding acquisition, Writing - review and editing. CS: Funding acquisition, Writing - review and editing. RV: Funding acquisition, Writing - review and editing. LQ: Funding acquisition, Writing - review and editing. AC: Funding acquisition, Writing - review and editing. MB: Funding acquisition, Writing - review and editing. GL: Conceptualization, Funding acquisition, Methodology, Supervision, Writing - original draft, Writing - review and editing. EN-L: Conceptualization, Funding acquisition, Methodology, Project administration, Supervision, Writing - original draft, Writing - review and editing.

## Footnotes

1
https://github.com/grosenberger/easypqp

2
https://www.qiagenbioinformatics.com/products/ingenuity-pathway-analysis
