# Supplementary material for: Clinical and pulmonary function analysis in long-COVID revealed that long-term pulmonary dysfunction is associated with vascular inflammation pathways and metabolic syndrome
Source: Front Med (Lausanne). 2023 Oct 6;10:1271863. doi: 10.3389/fmed.2023.1271863 (PMC10590130; doi:10.3389/fmed.2023.1271863)
Supplement: Supplementary file 1 [file Table_1.DOCX]

Supplementary Material

*Supplementary Figures*


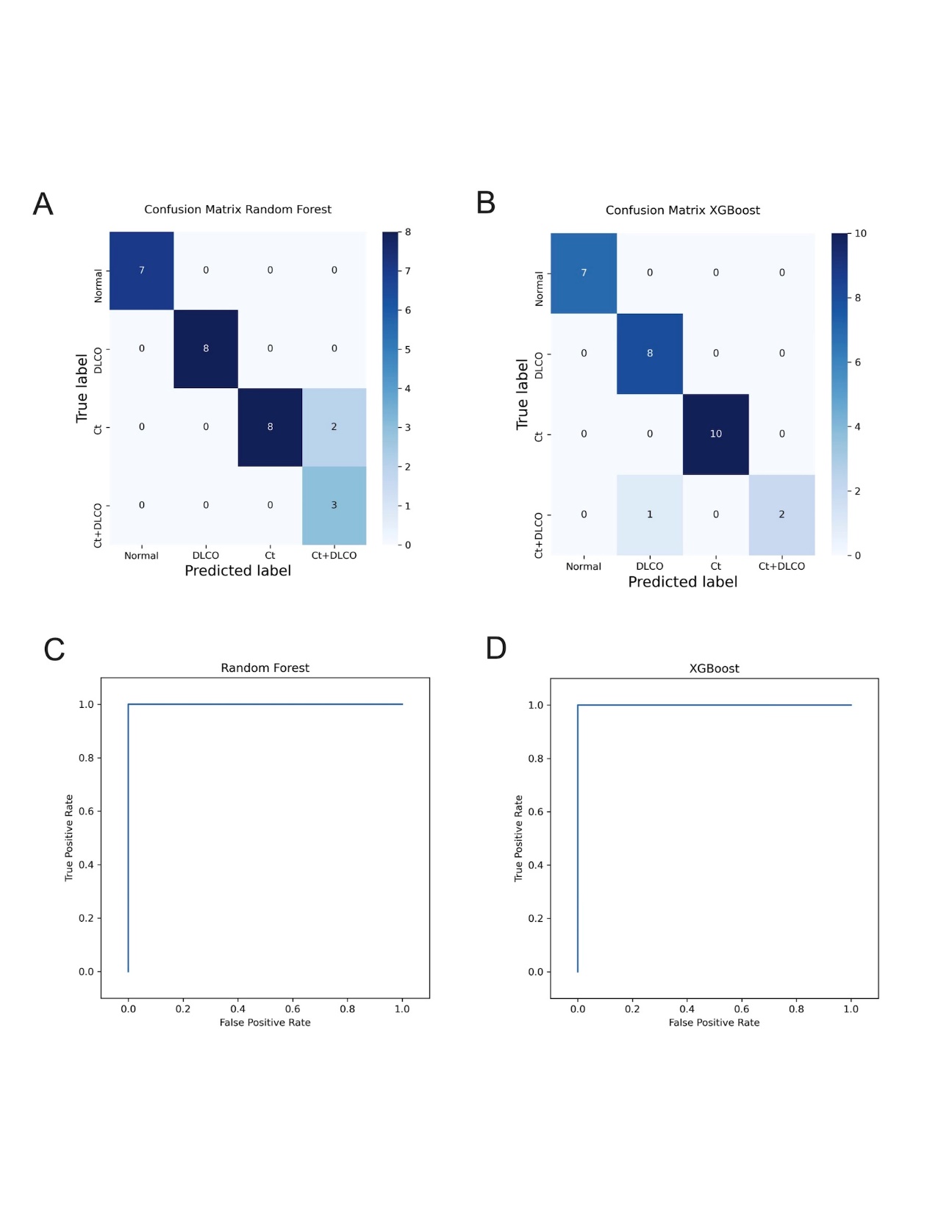


**Supplementary Figure 1: Classification all features.** Confusion matrix results for training and test data using **A)** Random Forest classifier and **B)** XGBoost classifier. AUC values for **C)** Random Forest classifier and **D)** XGBoost classifier.

**
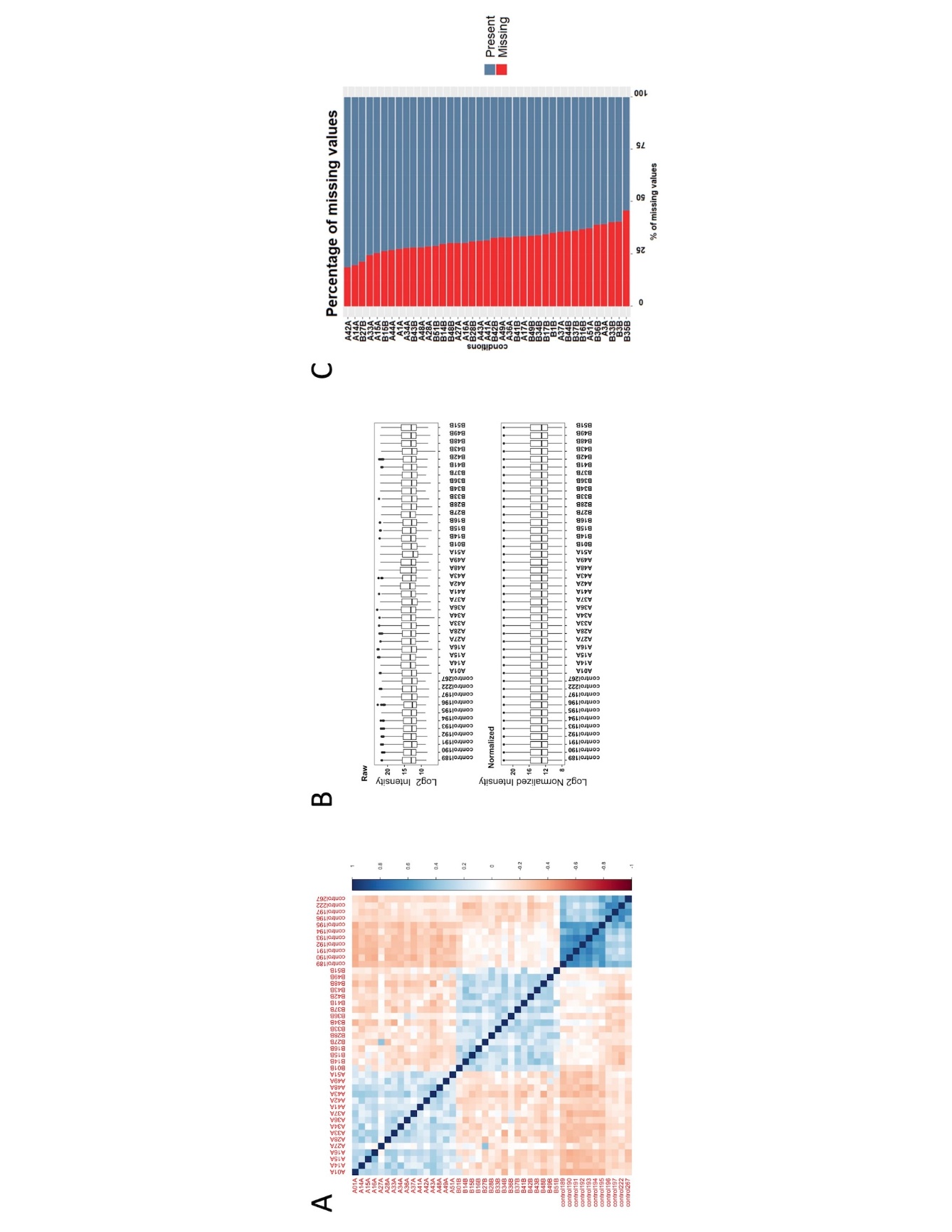
**

**Supplementary Figure 2:** **Quality control from proteomics analysis.** **A)** Heatmap from Pearson correlation data in the chromatography, **B)** normalized data, and **C)** Percentage of missing values.

**
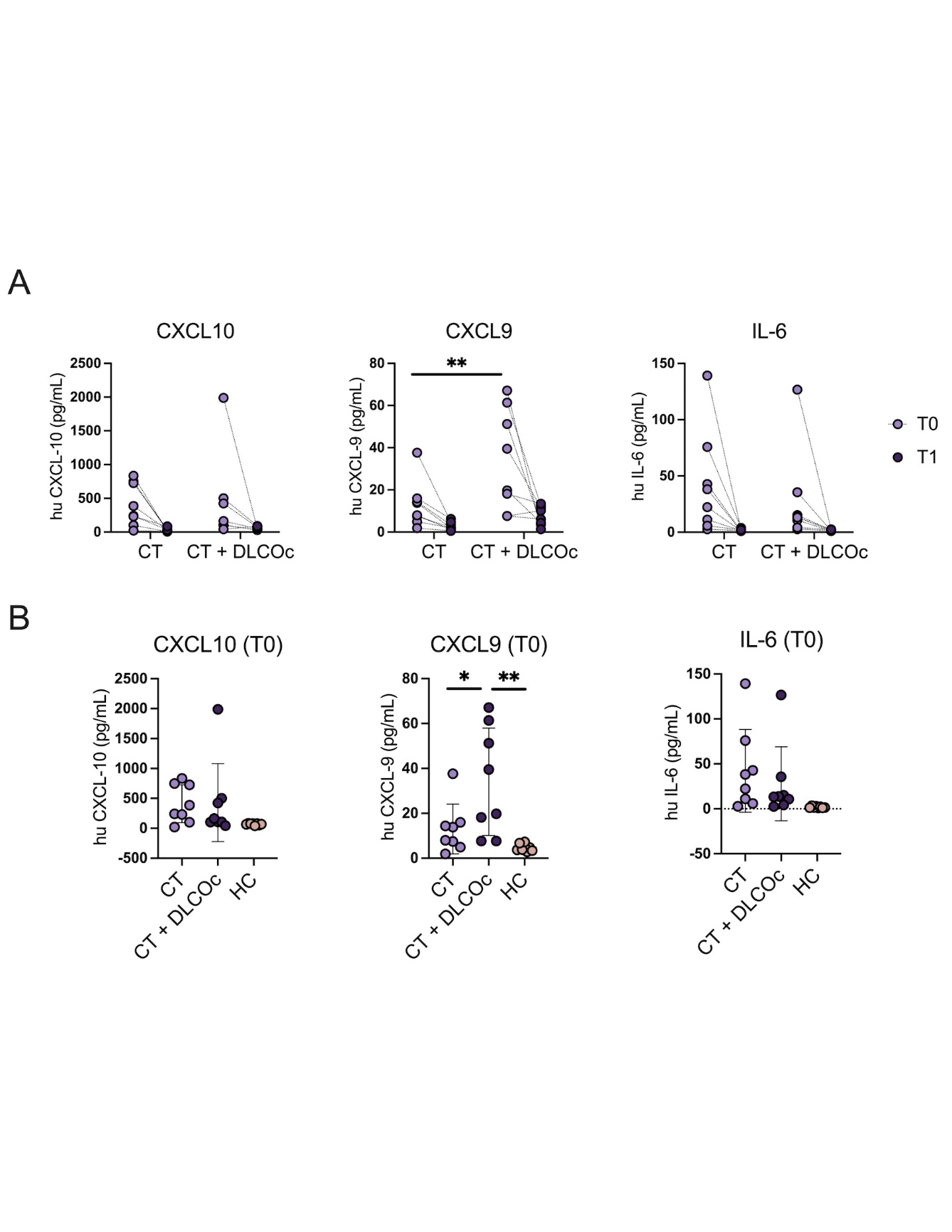
Supplementary Figure 3:** **CXCL10, CXCL9 and IL-6 levels between CT and CT+DLCOc groups during the acute phase.**

**A)** Symbols and lines plots of CXCL10, CXCL9 and IL-6 serum levels in CT and CT+DLCOc between the acute phase (T0) and the follow-up (T1) 2-way ANOVA tests with Sidak multiple comparison test; **p<0.01, *p<0.05. **B)** Scatter plots of CXCL10, CXCL9 and IL-6 levels between CT, CT+DLCOc and healthy controls during the acute phase (T0). Ordinary one-way ANOVA tests; **p<0.01, *p<0.05.

**
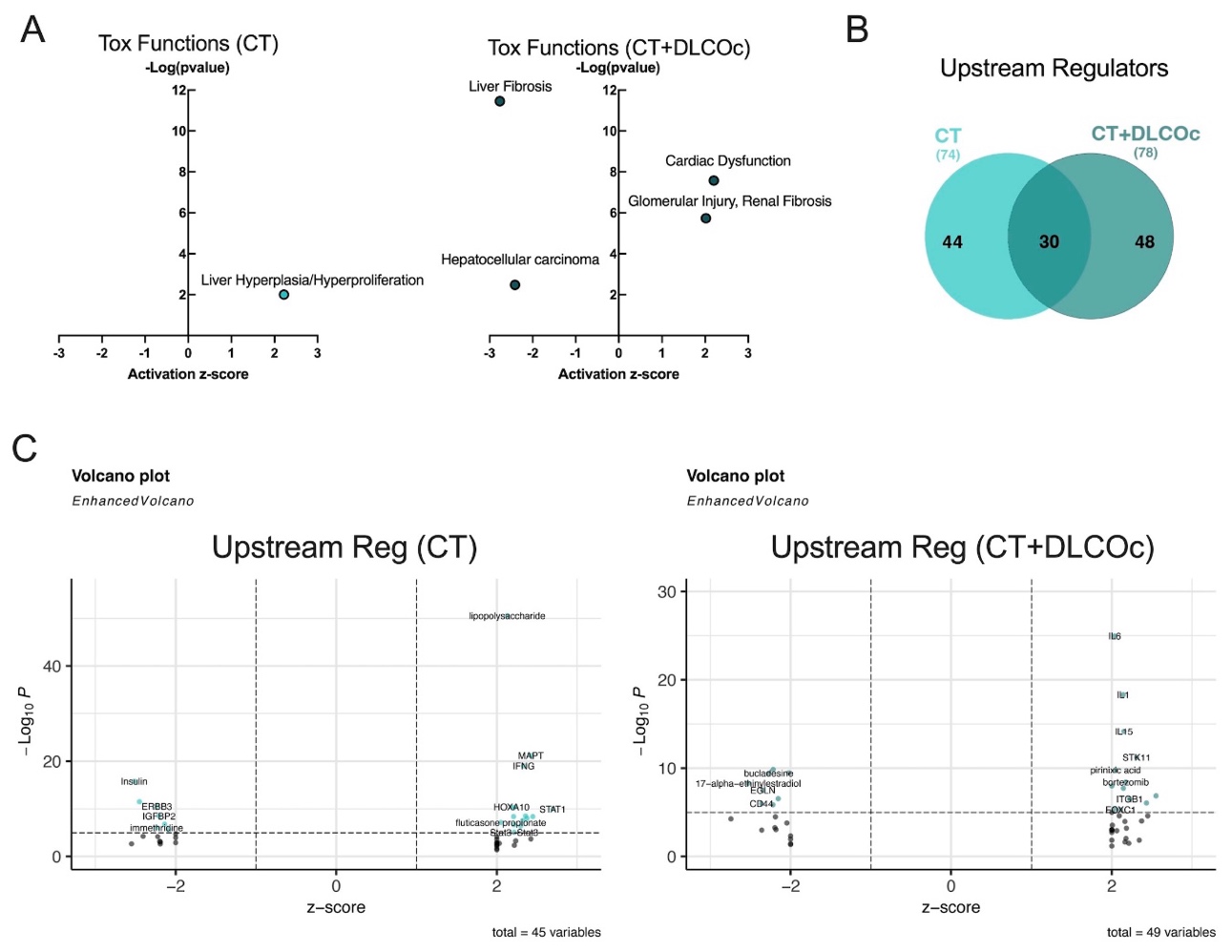
**

**Supplementary Figure 4:** **Tox Functions and upstream regulators.**

**A)** Significant Tox Functions inhibited (z-score < -2) or activated (z-score > 2) in CT and CT+DLCOc groups according to IPA analysis. **B)** Venn diagram of upstream regulators between CT and CT+DLCOc groups and **C)** CT or CT+DLCOc unique upstream regulators inhibited (z-score < -2) or activated (z-score > 2) according to IPA analysis.


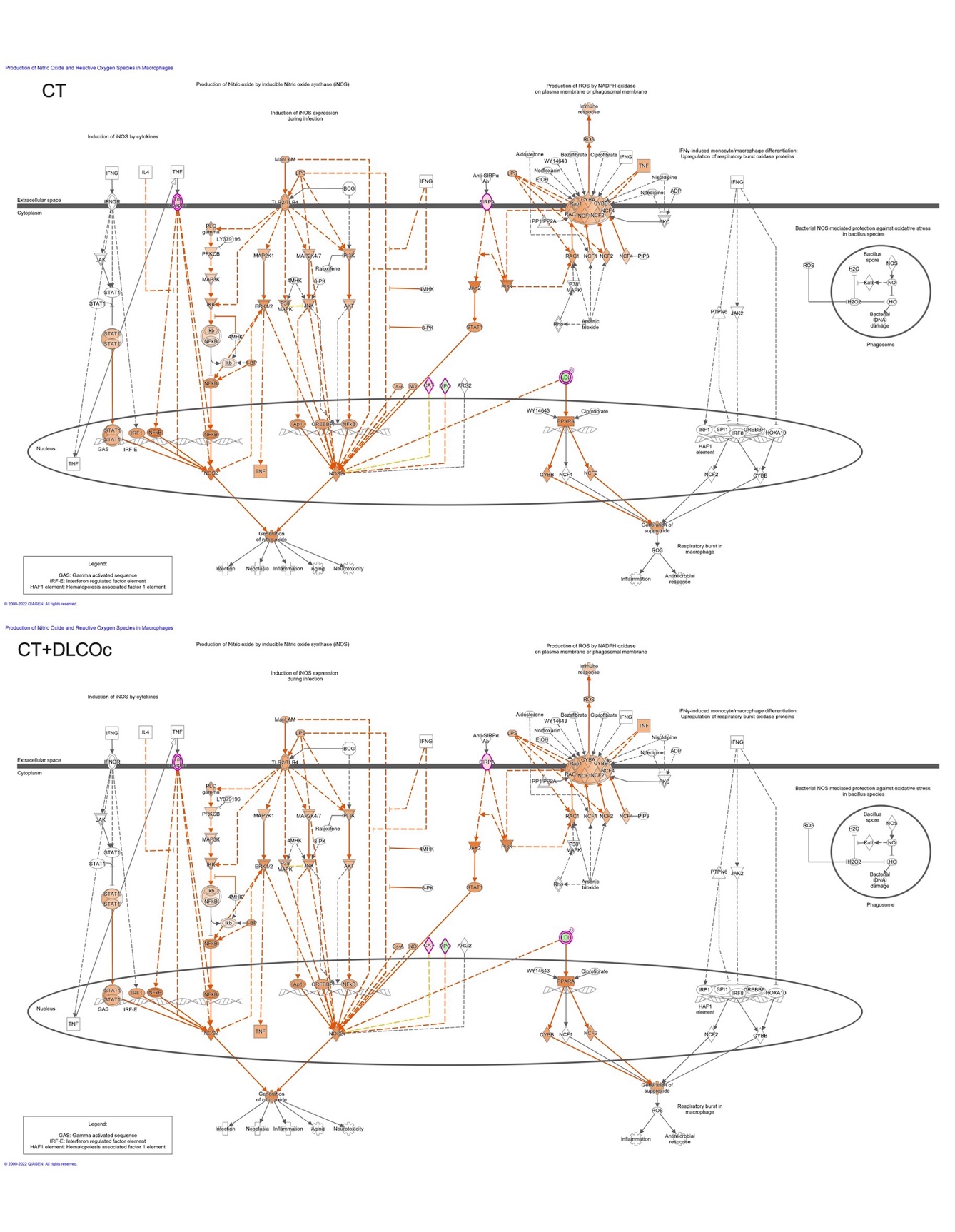


**Supplementary Figure 5:** **Production of Nitric Oxide and Reactive Oxygen Species in Macrophages.**

Ingenuity pathway analysis graphical representation of Production of Nitric Oxide and Reactive Oxygen Species in Macrophages in the CT (Top) and CT+DLCO (Bottom) groups at 4-months post-infection.

**
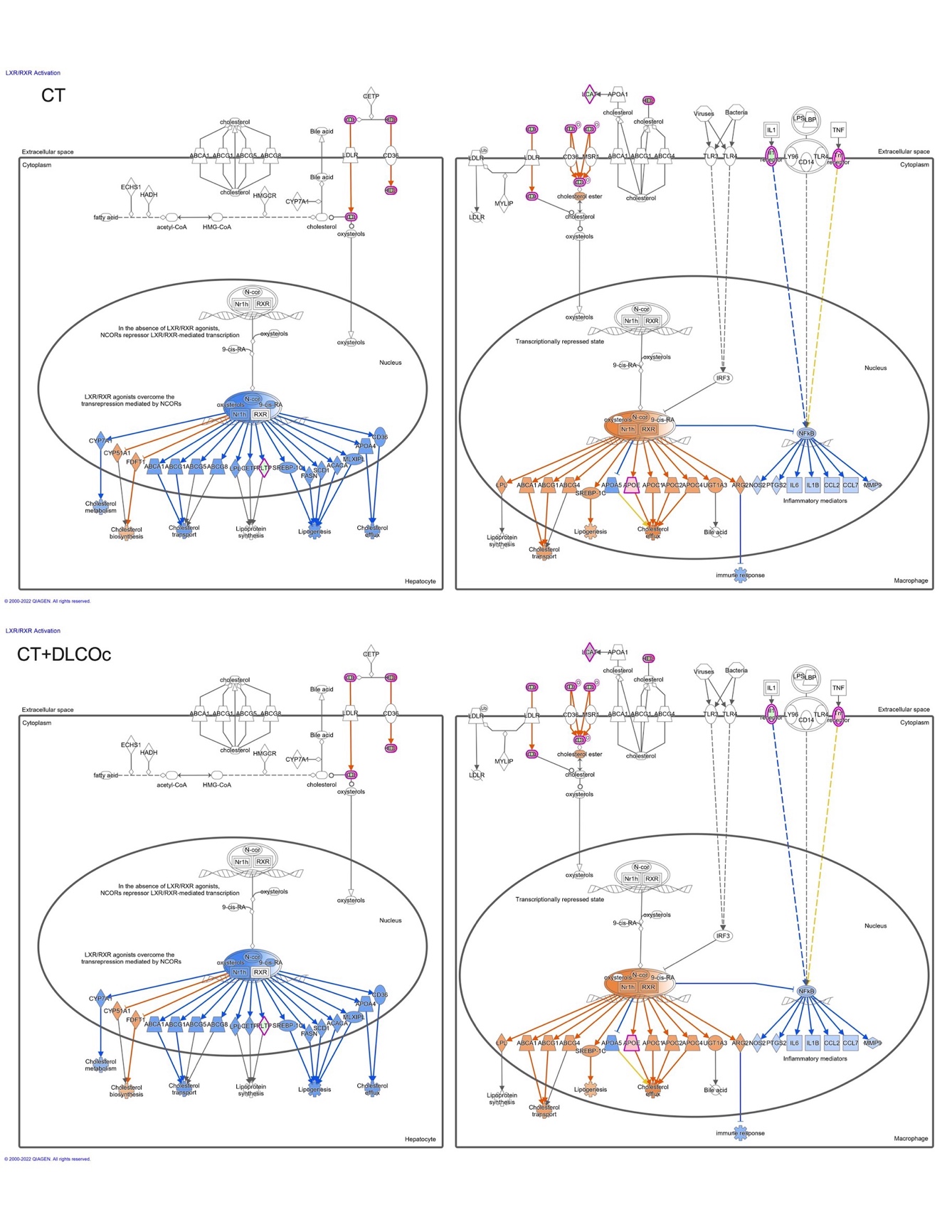
Supplementary Figure 6:** **LXR/RXR Activation pathway.**

Ingenuity pathway analysis graphical representation LXR/RXR Activation in the CT (Top) and CT+DLCO (Bottom) groups at 4-months post-infection.

*Supplementary Tables*

**Supplementary Table 1.** Total characteristic of COVID-19 subgroups for artificial intelligence analysis (n=60).

| **Feature (Mean ± SD)** | **CT**  **(n=23)** | **DLCOc**  **(n=5)** | **CT+DLCOc (n=14)** | **Normal**  **(n=18)** |
| --- | --- | --- | --- | --- |
| % Granulocytes | 62.70 ± 7.58 | 65.38 ± 6.72 | 61.77 ± 6.77 | 64.37 ± 8.35 |
| % Lymphocytes | 30.54 ± 6.52 | 28.6 ± 6.43 | 32.13 ± 6.36 | 28.90 ± 9.17 |
| % Monocytes | 6.59 ± 2.81 | 5.62 ± 1.28 | 6.09 ± 1.19 | 6.17 ± 1.54 |
| 6 MWT | 533.48 ± 93.60 | 498.4 ± 44.48 | 444 ± 143.16 | 575.72 ± 70.29 |
| Abdominal pain | 0.26 ± 0.44 | 0.2 ± 0.22 | 0.21 ± 0.42 | 0.50 ± 0.51 |
| Abnormal Chest CT | 1 ± 0 | 0 ± 0 | 1 ± 0 | 0 ± 0 |
| Abnormal DLCOc | 0 ± 0 | 1 ± 0 | 1 ± 0 | 0 ± 0 |
| Abnormal glucose | 0.73 ± 0.44 | 0.4 ± 0.54 | 0.5 ± 0.51 | 0.55 ± 0.51 |
| Abnormal handgrip | 0.82 ± 0.38 | 1 ± 0 | 1 ± 0 | 0.66 ± 0.48 |
| Abnormal HDL | 0.30 ± 0.47 | 0.2 ± 0.44 | 0.21 ± 0.42 | 0.33 ± 0.48 |
| Abnormal TG | 0.43 ± 0.50 | 0.2 ± 0.44 | 0.71 ± 0.46 | 0.44 ± 0.51 |
| Abnormal waist | 0.82 ± 0.38 | 0.8 ± 0.44 | 0.85 ± 0.36 | 0.55 ± 0.51 |
| ACE/AII | 0.21 ± 0.42 | 0 ± 0 | 0.5 ± 0.51 | 0 ± 0 |
| Acrophase | -3.97 ± 0.20 | -4.09 ± 0.30 | -4.14 ± 0.35 | -4.12 ± 0.28 |
| Afib | 0 ± 0 | 0 ± 0 | 0.07 ± 0.26 | 0 ± 0 |
| Age | 48.91 ± 10.25 | 44.8 ± 10.49 | 56.78 ± 11.94 | 35.61 ± 10.27 |
| Air trapping | 0.39 ± 0.50 | 0 ± 0 | 0.42 ± 0.51 | 0 ± 0 |
| Albumin | 4.30 ± 0.80 | 4.38 ± 0.77 | 4.2 ± 0.60 | 3.96 ± 0.80 |
| Alcohol | 0.65 ± 0.48 | 0.4 ± 0.54 | 0.64 ± 0.74 | 0.61 ± 0.50 |
| Alkaline phosphatase | 75.51 ± 23.12 | 73 ± 16.04 | 76.07 ± 15.99 | 63.65 ± 22.45 |
| Amplitude | 2287.10 ± 659.42 | 2415.27 ± 538.58 | 1982.05 ± 496.07 | 2724.72 ± 663.05 |
| Anosmia | 0.04 ± 0.20 | 0 ± 0 | 0.07 ± 0.26 | 0.11 ± 0.32 |
| ARDS | 0.78 ± 0.42 | 0 ± 0 | 0.85 ± 0.36 | 0.22 ± 0.42 |
| Arousal | 6.61 ± 4.29 | 6.8 ± 3.70 | 6.92 ± 2.92 | 8.92 ± 4.19 |
| Atelectasis | 0.13 ± 0.34 | 0 ± 0 | 0.21 ± 0.42 | 0.05 ± 0.23 |
| Ageusia | 0 ± 0 | 0 ± 0 | 0 ± 0 | 0.05 ± 0.23 |
| Average pulse | 70.34 ± 8.92 | 68 ± 5.78 | 72.14 ± 7.88 | 65.38 ± 5.86 |
| B cell Count | 155.29 ± 98.36 | 146.24 ± 44.81 | 153.84 ± 65.96 | 143.22 ± 88.79 |
| B cells | 8.83 ± 4.60 | 9.94 ± 2.63 | 7.62 ± 3.17 | 8.32 ± 3.09 |
| Beta-Blockers | 0 ± 0 | 0 ± 0 | 0.21 ± 0.42 | 0 ± 0 |
| Beck Depression | 11.60 ± 9.77 | 11.6 ± 6.46 | 10.64 ± 6.31 | 5.61 ± 6.10 |
| Bilirubin (direct) | 0.22 ± 0.10 | 0.25 ± 0.04 | 0.22 ± 0.12 | 0.24 ± 0.14 |
| Bilirubin (Indirect) | 0.51 ± 1.04 | 0.24 ± 0.07 | 0.27 ± 0.14 | 0.33 ± 0.16 |
| Bilirubin (Total) | 0.73 ± 1.07 | 0.50 ± 0.10 | 0.50 ± 0.26 | 0.58 ± 0.20 |
| Biological therapy | 0.09 ± 0.30 | 0 ± 0 | 0 ± 0 | 0 ± 0 |
| Body Mass Index | 30.91 ± 3.92 | 30.55 ± 4.85 | 32.80 ± 6.27 | 30.06 ± 5.14 |
| BNM | 0.52 ± 0.51 | 0 ± 0 | 0.46 ± 0.51 | 0.10 ± 0.31 |
| Borg basal | 1.26 ± 1.91 | 1.6 ± 2.19 | 2.28 ± 2.01 | 0.50 ± 1.20 |
| Borg final | 2.56 ± 2.37 | 3 ± 2.73 | 4.85 ± 2.82 | 2.44 ± 2.99 |
| Bronchiectasis | 0.08 ± 0.28 | 0 ± 0 | 0.28 ± 0.46 | 0 ± 0 |
| C3a | 54682.77 ± 37631.33 | 54320.12 ± 22041.43 | 46218.94 ± 16900.83 | 54769.96 ± 22963.86 |
| C4a | 293608.15 ± 270435.22 | 372082.14 ± 249595.17 | 2404729.27 ± 8103238.51 | 471897.98 ± 545240.07 |
| C5a | 3687.16 ± 1697.36 | 3893.78 ± 2344.75 | 5158.0192 ± 2053.22 | 3214.77 ± 2138.33 |
| Calcium | 8.156 ± 1.64 | 8.86 ± 1.29 | 9.42 ± 1.44 | 7.88 ± 2.46 |
| Calcium channel | 0.043 ± 0.20 | 0 ± 0 | 0.28 ± 0.46 | 0.05 ± 0.23 |
| Cancer | 0 ± 0 | 0 ± 0 | 0.07 ± 0.26 | 0 ± 0 |
| CCL2 | 49.16 ± 35.14 | 40.67 ± 15.59 | 42.48 ± 24.26 | 44.59 ± 29.79 |
| CCL5 | 481.51 ± 292.47 | 433.33 ± 230.86 | 325.43 ± 336.63 | 428.43 ± 324.82 |
| CD27- B cells (%) | 76.71 ± 7.37 | 79.3 ± 8.12 | 74.95 ± 8.26 | 74.82 ± 10.20 |
| CD27+ B cells (%) | 23.24 ± 7.33 | 20.7 ± 8.12 | 24.99 ± 8.23 | 25.12 ± 10.19 |
| CFI | 0.72 ± 0.06 | 0.71 ± 0.03 | 0.71 ± 0.05 | 0.74 ± 0.05 |
| Chalder total points | 15.91 ± 7.53 | 15.6 ± 10.26 | 20.78 ± 7.93 | 12.50 ± 9.10 |
| Change smell | 0.43 ± 0.50 | 0.6 ± 0.54 | 0.35 ± 0.49 | 0.38 ± 0.50 |
| Change in HrQOL | 0.73 ± 0.44 | 0.8 ± 0.44 | 0.71 ± 0.46 | 0.50 ± 0.51 |
| Change taste | 0.39 ± 0.49 | 0.6 ± 0.54 | 0.28 ± 0.46 | 0.44 ± 0.51 |
| CHCM | 35.16 ± 0.76 | 35.86 ± 0.90 | 34.70 ± 0.53 | 35.47 ± 0.86 |
| CHD | 0 ± 0 | 0 ± 0 | 0 ± 0 | 0 ± 0 |
| Chest CT | 0.95 ± 0.20 | 0.8 ± 0.44 | 1 ± 0 | 0.77 ± 0.42 |
| Chest pain | 0.43 ± 0.50 | 0.4 ± 0.54 | 0.57 ± 0.51 | 0.38 ± 0.50 |
| Cyanosis | 0 ± 0 | 0 ± 0 | 0 ± 0 | 0 ± 0 |
| CK total | 164.97 ± 72.23 | 189.92 ± 63.07 | 185.78 ± 93.63 | 227.28 ± 154.60 |
| CKD | 0 ± 0 | 0 ± 0 | 0 ± 0 | 0 ± 0 |
| CNAF | 0.28 ± 0.46 | 0 ± 0 | 0.84 ± 0.37 | 0 ± 0 |
| Consolidation | 0 ± 0 | 0 ± 0 | 0.07 ± 0.26 | 0 ± 0 |
| COPD | 0 ± 0 | 0 ± 0 | 0 ± 0 | 0 ± 0 |
| Corticosteroids | 0.57 ± 0.50 | 0 ± 0 | 0.92 ± 0.27 | 0.10 ± 0.31 |
| Cough | 0.69 ± 0.47 | 0.4 ± 0.54 | 0.71 ± 0.46 | 0.61 ± 0.50 |
| Creatinemia | 0.92 ± 0.24 | 0.80 ± 0.23 | 0.99 ± 0.41 | 0.78 ± 0.25 |
| CXCL10 | 100.49 ± 44.61 | 87.99 ± 36.83 | 136.28 ± 54.18 | 74.61 ± 40.22 |
| CXCL9 | 15.36 ± 11.77 | 14.89 ± 15.52 | 30.01 ± 21.58 | 11.84 ± 13.81 |
| Days in ICU | 9.85 ± 9.10 | 0 ± 0 | 9.66 ± 10.56 | 4.30 ± 5.18 |
| Days in IVM | 7.71 ± 8.30 | 0 ± 0 | 7.66 ± 8.24 | 2.70 ± 4.52 |
| Days in hospital | 20.12 ± 21.89 | 0 ± 0 | 26.63 ± 22.49 | 7.22 ± 6.68 |
| DBP | 78.56 ± 13.83 | 69.6 ± 11.39 | 75.35 ± 13.14 | 73.50 ± 13.90 |
| DBP>90 | 0.34 ± 0.48 | 0 ± 0 | 0.14 ± 0.36 | 0.16 ± 0.38 |
| Desaturation | 0.08 ± 0.28 | 0 ± 0 | 0 ± 0 | 0.05 ± 0.23 |
| D-dimer | 1628. 68 ± 1209.79 |  | 1350.4 ± 874.52 | 1194.62 ± 1209.96 |
| Diarrhea | 0.34 ± 0.48 | 0.4 ± 0.54 | 0.21 ± 0.42 | 0.44 ± 0.51 |
| DLCO/VA | 90.81 ± 17.61 | 71 ± 3.16 | 70.07 ± 9.56 | 88.77 ± 14.78 |
| DLCO<80 | 0 ± 0 | 0.6 ± 0.54 | 0.85 ± 0.36 | 0 ± 0 |
| DLCO corrected | 97.86 ± 14.12 | 75.2 ± 2.38 | 63.35 ± 13.83 | 101.72 ± 17.02 |
| Drop >3% | 0.17 ± 0.38 | 0 ± 0 | 0.57 ± 0.51 | 0.16 ± 0.38 |
| Drop in Spo2 | -0.21 ± 1.27 | -0.8 ± 1.30 | 0.64 ± 1.69 | -0.11 ± 0.90 |
| Dyspnea | 0.78 ± 0.42 | 0.2 ± 0.44 | 1 ± 0 | 0.61 ± 0.50 |
| Erythrocytes | 4.68 ± 0.45 | 4.56 ± 0.29 | 4.54 ± 0.48 | 4.86 ± 0.46 |
| ESS | 7.78 ± 4.73 | 7.6 ± 2.60 | 8.42 ± 0.51 | 9.05 ± 5.70 |
| Fatigue | 0.78 ± 0.42 | 0.6 ± 0.54 | 0.85 ± 0.36 | 0.61 ± 0.50 |
| Fatigue (Chalder) | 5.30 ± 2.51 | 5.2 ± 3.42 | 6.92 ± 2.64 | 4.16 ± 3.03 |
| Fatigue basal | 1.56 ± 2.21 | 2.8 ± 2.58 | 2.85 ± 2.17 | 0.61 ± 1.75 |
| Fatigue final | 2.65 ± 2.65 | 3.4 ± 2.88 | 5.5 ± 2.40 | 2.55 ± 2.95 |
| Ferritin | 2179.05 ± 1719.32 |  | 2372.66 ± 1409.36 | 1267.14 ± 1431.10 |
| FEV1<70% | 0.21 ± 0.42 | 0 ± 0 | 0.21 ± 0.42 | 0.11 ± 0.32 |
| Fever | 0.65 ± 0.48 | 0.6 ± 0.54 | 0.64 ± 0.49 | 0.50 ± 0.51 |
| Fibrinogen | 631.46 ± 135.22 |  | 797.77 ± 313.84 | 679 ± 208.16 |
| Fibrotic lesions | 0.13 ± 0.34 | 0 ± 0 | 0.28 ± 0.46 | 0.05 ± 0.23 |
| GB | 11742 ± 4883.08 |  | 10521.66 ± 5916.70 | 8355.11 ± 4467.11 |
| GGT | 34.12 ± 36.96 | 31.1 ± 24.15 | 38.5 ± 36.75 | 31.88 ± 29.59 |
| Globulin | 2.83 ± 0.58 | 2.86 ± 0.23 | 2.92 ± 0.56 | 2.52 ± 0.55 |
| Glucose | 122.29 ± 35.50 | 103.2 ± 21.42 | 115.85 ± 53.51 | 109.83 ± 53.15 |
| GOT (AST) | 22.05 ± 11.94 | 22.66 ± 2.99 | 22.28 ± 12.60 | 26.47 ± 17.90 |
| GPT (ALT) | 30.15 ± 24.54 | 27.78 ± 11.48 | 24.21 ± 16.21 | 33.46 ± 26.19 |
| Grade ARDS | 0.78 ± 0.42 | 0 ± 0 | 0.85 ± 0.36 | 0.22 ± 0.42 |
| Granulocytes | 3716.65 ± 1282.92 | 3624.12 ± 1515.02 | 4084.72 ± 1136.45 | 3837.33 ± 1012.53 |
| Grond-glass | 0.61 ± 0.50 | 0 ± 0 | 0.92 ± 0.26 | 0 ± 0 |
| HADS-A | 7.34 ± 4.97 | 7.8 ± 4.86 | 6.64 ± 3.81 | 4.38 ± 3.03 |
| HADS-D | 4.78 ± 3.89 | 9 ± 3.93 | 4.71 ± 3.19 | 3.05 ± 3.29 |
| HCM | 31.73 ± 1.69 | 32.98 ± 0.56 | 31.36 ± 1.33 | 31.81 ± 1.33 |
| HCO3 | 23.13 ± 1.42 | 23.82 ± 1.78 | 24.06 ± 1.36 | 23.62 ± 1.97 |
| HDL-cholesterol | 49.50 ± 19.61 | 61.89 ± 20.31 | 57.21 ± 17.84 | 48.52 ± 16.29 |
| Headache | 0.60 ± 0.49 | 0.8 ± 0.44 | 0.57 ± 0.51 | 0.61 ± 0.50 |
| Heart rate | 78.52 ± 11.34 | 71.8 ± 5.21 | 79.14 ± 12.93 | 77.72 ± 10.18 |
| Height | 1.66 ± 0.08 | 1.58 ± 0.12 | 1.59 ± 0.09 | 10.69 ± 38.25 |
| Hematocrit | 42.05 ± 3.23 | 41.98 ± 2.86 | 41.02 ± 3.82 | 43.63 ± 3.60 |
| Hemoglobin | 14.80 ± 1.26 | 15.06 ± 1.15 | 14.23 ± 1.31 | 15.46 ± 1.44 |
| Hip circumference | 108.95 ± 8.46 | 108.2 ± 9.67 | 112.42 ± 9.83 | 105.11 ± 9.80 |
| HMW | 5.52 ± 4.25 | 5.21 ± 2.85 | 4.35 ± 2.09 | 4.72 ± 2.54 |
| HOMA | 11.01 ± 23.57 | 4.21 ± 1.57 | 8.62 ± 8.05 | 3.71 ± 1.75 |
| HR (basal) | 82.69 ± 13.40 | 77.6 ± 11.84 | 81 ± 11.92 | 73.44 ± 10.99 |
| HR (final) | 103.69 ± 18.58 | 93.4 ± 22.04 | 103.92 ± 21.08 | 100.44 ± 23.69 |
| HTA | 0.30 ± 0.47 | 0 ± 0 | 0.64 ± 0.49 | 0.22 ± 0.42 |
| Hypolipemic drug | 0.17 ± 0.38 | 0.2 ± 0.44 | 0.35 ± 0.49 | 0.11 ± 0.32 |
| Hypothyroidism | 0.08 ± 0.28 | 0.2 ± 0.44 | 0.14 ± 0.36 | 0 ± 0 |
| IgG SARS-COV-2 | 15.47 ± 9.45 | 8.52 ± 5.96 | 17.33 ± 9.07 | 7.98 ± 5.16 |
| IgM SARS-COV-2 | 1.21 ± 2.53 | 1.75 ± 1.97 | 2.31 ± 3.07 | 1.64 ± 2.05 |
| IL-8 | 6.814 ± 5.19 | 5.15 ± 1.71 | 6.08 ± 3.24 | 4.34 ± 3.03 |
| IL-12 | 0.57 ± 0.14 | 0.60 ± 0.09 | 0.63 ± 0.16 | 0.56 ± 0.09 |
| IL-1b | 0.23 ± 0.19 | 0.28 ± 0.24 | 0.43 ± 0.79 | 0.41 ± 0.67 |
| IL-6 | 2.11 ± 1.01 | 1.80 ± 0.67 | 2.21 ± 0.96 | 1.44 ± 0.65 |
| Insomnia (ISI) | 10.30 ± 7.31 | 15 ± 6.28 | 9.64 ± 6.27 | 8.55 ± 5.56 |
| Insulin | 29.40 ± 7.31 | 16.36 ± 4.96 | 28 ± 20.37 | 14.57 ± 6.84 |
| Insulin resistance | 0.21 ± 0.42 | 0 ± 0 | 0.42 ± 0.51 | 0 ± 0 |
| Interdaily stability | 0.44 ± 0.07 | 0.46 ± 0.06 | 0.47 ± 0.10 | 0.47 ± 0.08 |
| Interlobular thickening | 0.17 ± 0.38 | 0 ± 0 | 0.71 ± 0.46 | 0 ± 0 |
| Intradaily stability | 0.66 ± 0.10 | 0.62 ± 0.08 | 0.71 ± 0.11 | 0.60 ± 0.10 |
| IRSS | 0.17 ± 0.38 | 0.2 ± 0.44 | 0.07 ± 0.26 | 0 ± 0 |
| L5 | 58.35 ± 30.89 | 75.40 ± 75.88 | 82.41 ± 33.82 | 74.02 ± 31.09 |
| LDH | 198.21 ± 80.84 | 186.20 ± 30.32 | 192.71 ± 56.31 | 187.98 ± 95.74 |
| LDL cholesterol | 114.70 ± 41.57 | 132.48 ± 31 | 117.16 ± 51.12 | 110.53 ± 37.05 |
| Leucocytes | 5873.91 ± 1671.74 | 5420 ± 1755.56 | 6550 ± 1455.36 | 5950 ± 1270.59 |
| Lymphocyte | 887.75 ± 338.95 |  | 747.58 ± 336.55 | 1436.77 ± 844.17 |
| Lowest O2 | 83.13 ± 5.52 | 86.4 ± 4.03 | 81 ± 6.21 | 86.33 ± 7.12 |
| Lymph | 1777.66 ± 580.13 | 1480.44 ± 281.96 | 2063.30 ± 464.33 | 1716.99 ± 641.19 |
| M10 | 6160.82 ± 1394.63 | 5737.03 ± 1195.72 | 5573.60 ± 1410.88 | 7020.24 ± 1109.04 |
| Max pulse | 103.08 ± 10.87 | 94.6 ± 4.50 | 107.21 ± 15.86 | 98.77 ± 9.21 |
| Mean O2 | 93.95 ± 1.77 | 94.8 ± 0.44 | 93.35 ± 1.78 | 94.83 ± 1.50 |
| MEQ | 57.91 ± 6.25 | 49.80 ± 8.58 | 57.21 ± 9.84 | 56.41 ± 8.83 |
| MEQ (chronotype) | 1.47 ± 0.51 | 1 ± 0.70 | 1.35 ± 0.63 | 1.52 ± 0.71 |
| Mesor | 2782.69 ± 520.09 | 2633.86 ± 312.91 | 2647.83 ± 979.99 | 3196.90 ± 679.18 |
| Metformin | 0.39 ± 0.49 | 0 ± 0 | 0.42 ± 0.51 | 0.05 ± 0.23 |
| Min pulse | 57.52 ± 8.05 | 55.40 ± 8.79 | 55.92 ± 8.48 | 50.61 ± 6.57 |
| Mixed ground-glass | 0.08 ± 0.28 | 0 ± 0 | 0.07 ± 0.26 | 0 ± 0 |
| mMRC | 0.86 ± 0.45 | 0.6 ± 0.54 | 1.28 ± 0.82 | 0.5 ± 0.51 |
| Monocytes | 373.67 ± 163.87 | 293.84 ± 75.22 | 401.97 ± 147.42 | 365.75 ± 115.71 |
| Myalgia | 0.56 ± 0.50 | 0.8 ± 0.44 | 0.5 ± 0.51 | 0.83 ± 0.38 |
| NAFLD | 0.04 ± 0.20 | 0.20 ± 0.44 | 0.21 ± 0.42 | 0.11 ± 0.32 |
| Neck circumference | 41.39 ± 4.47 | 41.20 ± 7.01 | 43.5 ± 5.85 | 41.38 ± 5.29 |
| Nonsolid nodule | 0.17 ± 0.38 | 0 ± 0 | 0.35 ± 0.49 | 0 ± 0 |
| Number of Lobes affected | 2.13 ± 1.28 | 0 ± 0 | 2.07 ± 1.38 | 0.27 ± 0.66 |
| ODI | 11.03 ± 10.12 | 10.72 ± 15.14 | 13.39 ± 12.65 | 6.43 ± 9.10 |
| Odynophagia | 0.08 ± 0.28 | 0.20 ± 0.44 | 0.07 ± 0.26 | 0.05 ± 0.23 |
| Onset latency | 2.38 ± 2.41 | 1 ± 0 | 2 ± 1.77 | 2.28 ± 1.54 |
| Pack/year | 3.13 ± 6.84 | 2.6 ± 4.21 | 5.21 ± 13.51 | 1.88 ± 4.95 |
| Pafi | 200.55 ± 64.82 | 360 ± 0 | 178.07 ± 45.68 | 271.44 ± 79.22 |
| pAO2 | 91.01 ± 4.93 | 89.39 ± 2.94 | 87.23 ± 3.33 | 89.94 ± 3.99 |
| pCO2 | 38.36 ± 3.25 | 39.2 ± 2.35 | 40.92 ± 2.66 | 38.75 ± 3.19 |
| PCR | 144.20 ± 113.50 | NA | 172.94 ± 114.20 | 143.86 ± 155.62 |
| PEF | 480.17 ± 151.45 | 382.6 ± 52.52 | 365.64 ± 126.63 | 482.44 ± 120.43 |
| pH | 7.39 ± 0.02 | 7.39 ± 0.01 | 7.39 ± 0.01 | 7.39 ± 0.01 |
| Phosphorous | 3.97 ± 0.79 | 3.68 ± 0.64 | 4 ± 0.58 | 3.38 ± 0.83 |
| Pittsburg | 10 ± 4.67 | 12.8 ± 1.30 | 10.92 ± 5.03 | 8.16 ± 4.43 |
| Platelets | 256782.60 ± 37182.54 | 303600 ± 63030.15 | 310142.85 ± 59863.76 | 277388.88 ± 64142.43 |
| pO2 | 99.38 ± 10.55 | 99.57 ± 8.11 | 96.51 ± 7.38 | 104.50 ± 8.43 |
| Polypnea | 0.69 ± 0.47 | 0.20 ± 0.44 | 0.78 ± 0.42 | 0.44 ± 0.51 |
| postEuroQoL | 64.34 ± 24.13 | 65 ± 29.58 | 66.42 ± 22.39 | 77.22 ± 18.40 |
| postFEF 25-75% (L/s) % Teo | 111.82 ± 31.38 | 102.40 ± 29.28 | 107.57 ± 49.86 | 97.33 ± 24.23 |
| postFEF 25-75% (L/s)real | 3.63 ± 1.15 | 3.02 ± 0.73 | 2.69 ± 1.54 | 3.66 ± 1.06 |
| postFEF max (L/s) Teo | 92.60 ± 25.08 | 88.60 ± 35.23 | 68.64 ± 26.59 | 86.44 ± 30.99 |
| postFEF max (L/s) real | 7.75 ± 2.69 | 5.66 ± 1.18 | 4.65 ± 2.17 | 6.93 ± 2.50 |
| postFEV1 (L) % Teo | 94.65 ± 14.61 | 99.4 ± 11.63 | 83.07 ± 15.71 | 98.72 ± 16.98 |
| postFEV1 (L) real | 3.05 ± 0.56 | 2.85 ± 0.76 | 2.28 ± 0.59 | 3.52 ± 0.92 |
| postFVC (L) (%) | 88.34 ± 16.03 | 91.8 ± 13.82 | 77.28 ± 11.98 | 98.72 ± 18.32 |
| postFVC (L) real | 3.56 ± 0.76 | 3.19 ± 0.58 | 2.63 ± 0.63 | 4.25 ± 1.20 |
| postVEF1/FVC real (%) | 87.43 ± 7.24 | 88.60 ± 10.06 | 85.28 ± 8.65 | 83.44 ± 6.35 |
| postVEF1/FVC % Teo | 107.26 ± 8.78 | 108 ± 11.81 | 106.85 ± 10.70 | 100.27 ± 7.25 |
| Potassium sparing | 0 ± 0 | 0 ± 0 | 0.07 ± 0.26 | 0 ± 0 |
| preEuroQoL | 88.47 ± 11.81 | 93 ± 6.70 | 87.5 ± 11.22 | 91.33 ± 8.31 |
| preFEF 25-75% (L/s) real | 3.50 ± 1.10 | 2.60 ± 0.69 | 10.21 ± 27.31 | 3.56 ± 0.99 |
| preFEF 25-75% % Teo | 111.13 ± 31.85 | 87.60 ± 25.05 | 108.28 ± 44.05 | 94.61 ± 20.42 |
| preFEF max (L/s) % Teo | 93.21 ± 23.39 | 72.80 ± 40.57 | 74.64 ± 25.06 | 86.38 ± 28.28 |
| preFEF max (L/s) real | 7.59 ± 2.49 | 4.78 ± 1.59 | 5.24 ± 1.90 | 7.08 ± 2.59 |
| preFEV1 (L) % Teo | 94.56 ± 14.18 | 91.6 ± 19.59 | 84.85 ± 16.67 | 98.22 ± 14.98 |
| preFEV1 (L) real | 3.04 ± 0.52 | 2.65 ± 0.24 | 2.39 ± 0.61 | 3.49 ± 0.86 |
| preFVC (L) % Teo | 89.78 ± 14.41 | 90.6 ± 14.80 | 78.57 ± 12.67 | 98.72 ± 16.01 |
| preFVC (L) real | 3.61 ± 0.69 | 3.02 ± 0.66 | 2.63 ± 0.71 | 4.27 ± 1.16 |
| preVEF1/FVC % Teo | 105.73 ± 9.12 | 99.60 ± 11.30 | 107 ± 9.87 | 99.16 ± 6.77 |
| preVEF1/FVC real (%) | 85.26 ± 7.21 | 82.20 ± 9.41 | 85.28 ± 7.68 | 82.77 ± 6.28 |
| Prono | 0.42 ± 0.50 | 0 ± 0 | 0.30 ± 0.48 | 0.10 ± 0.31 |
| Prono Vigil | 0.33 ± 0.48 | 0 ± 0 | 0.76 ± 0.43 | 0.30 ± 0.48 |
| Protein Total (Prot Tot) | 7.13 ± 1.25 | 7.24 ± 0.79 | 7.12 ± 1.07 | 6.48 ± 1.15 |
| RDI | 11.78 ± 9.84 | 11.40 ± 13.95 | 14.57 ± 11.25 | 6.72 ± 7.54 |
| RDI > 15 | 0.30 ± 0.47 | 0.20 ± 0.44 | 0.35 ± 0.49 | 0.11 ± 0.32 |
| RDI > 5 | 0.73 ± 0.44 | 0.60 ± 0.54 | 0.78 ± 0.42 | 0.33 ± 0.48 |
| RDI > 30 | 0.04 ± 0.20 | 0.20 ± 0.44 | 0.07 ± 0.26 | 0 ± 0 |
| Record time | 335.34 ± 101.89 | 350.60 ± 79.56 | 424.14 ± 126.85 | 322.94 ± 112.20 |
| Real A/G | 1.54 ± 0.24 | 1.54 ± 0.30 | 1.47 ± 0.24 | 1.59 ± 0.31 |
| Reticular lesions | 0.04 ± 0.20 | 0 ± 0 | 0.14 ± 0.36 | 0.05 ± 0.23 |
| Rural area | 0.17 ± 0.38 | 0 ± 0 | 0.14 ± 0.36 | 0 ± 0 |
| SATED | 6.13 ± 2.30 | 6.40 ± 2.88 | 5.14 ± 2.74 | 6.05 ± 2.57 |
| SBP | 135.34 ± 17.14 | 123.40 ± 17.44 | 135.14 ± 19.96 | 125.66 ± 17.24 |
| SBP/DBP | 0.60 ± 0.49 | 0.40 ± 0.54 | 0.85 ± 0.36 | 0.38 ± 0.50 |
| SBP > 140 | 0.34 ± 0.48 | 0.40 ± 0.54 | 0.28 ± 0.46 | 0.16 ± 0.38 |
| Scholar | 1.04 ± 0.92 | 1.40 ± 0.89 | 0.42 ± 0.75 | 1.33 ± 0.76 |
| SD arousal | 4.09 ± 2.38 | 3.40 ± 2.79 | 3.61 ± 1.75 | 4.35 ± 1.90 |
| SD onset | 3.19 ± 3.73 | 1.60 ± 0.54 | 2.92 ± 3.06 | 3 ± 3.11 |
| SD sleep eff | 9.52 ± 8.23 | 6.51 ± 4.86 | 8.60 ± 3.68 | 9.27 ± 8.28 |
| SD TNS | 154.80 ± 67.15 | 165 ± 75.54 | 170.92 ± 50.86 | 154.14 ± 61.80 |
| SD TST | 150.23 ± 80.72 | 149.60 ± 75.54 | 155.92 ± 46.32 | 131.78 ± 60.78 |
| SD WASO | 29.57 ± 17.23 | 26 ± 17.08 | 26.46 ± 9.89 | 31.57 ± 10.32 |
| SF-12 mental score | 45079.21 ± 10268.89 | 41034.8 ± 13157.04 | 49146.5 ± 12280.98 | 45122.26 ± 14950.40 |
| SF-physical score | 40609.65 ± 13008.23 | 46464.6 ± 11165.04 | 35127.85 ± 8971.97 | 50347.83 ± 7890.02 |
| Sleep efficiency | 87.63 ± 6.86 | 90.30 ± 6.72 | 85.52 ± 5.61 | 85.71 ± 7.42 |
| Smet (point) | 2.91 ± 1.04 | 2 ± 1 | 3.14 ± 1.02 | 2.27 ± 1.40 |
| Smet (yes/no) | 0.65 ± 0.48 | 0.40 ± 0.54 | 0.71 ± 0.46 | 0.50 ± 0.51 |
| Smoker | 0.69 ± 0.87 | 1 ± 1 | 0.64 ± 0.92 | 0.44 ± 0.51 |
| Solid nodule | 0.26 ± 0.44 | 0 ± 0 | 0.35 ± 0.49 | 0 ± 0 |
| Sore throat | 0.34 ± 0.48 | 0.80 ± 0.44 | 0.42 ± 0.51 | 0.44 ± 0.51 |
| SpO2 | 97.39 ± 1.58 | 97.80 ± 2.04 | 97.35 ± 1.86 | 96.5 ± 2.03 |
| SpO2 (basal) | 98 ± 1.16 | 98.40 ± 1.51 | 96.71 ± 2.12 | 98.22 ± 1.51 |
| Spo2 (final) | 98.21 ± 1.34 | 99.2 ± 0.44 | 96.07 ± 2.58 | 98.33 ± 1.57 |
| SQALI | 62.21 ± 21.15 | 60 ± 20.33 | 61.28 ± 20.63 | 75.23 ± 14.43 |
| Stop Bang | 3.34 ± 1.87 | 2.6 ± 1.67 | 4.21 ± 1.88 | 2.22 ± 1.62 |
| Stroke | 0 ± 0 | 0 ± 0 | 0.07 ± 0.26 | 0 ± 0 |
| T2DM | 0.17 ± 0.38 | 0 ± 0 | 0.14 ± 0.36 | 0.05 ± 0.23 |
| T90 | 10.13 ± 21.99 | 0.6 ± 1.34 | 14.1 ± 17.56 | 2.33 ± 4.86 |
| Thiazide drug | 0.04 ± 0.20 | 0 ± 0 | 0.21 ± 0.42 | 0 ± 0 |
| Thorax pain | 0.04 ± 0.20 | 0 ± 0 | 0.14 ± 0.36 | 0.05 ± 0.23 |
| TNF-a | 0.33 ± 0.38 | 0.15 ± 0.14 | 0.57 ± 0.52 | 0.49 ± 0.26 |
| TNS | 426.19 ± 105.30 | 418.80 ± 83.24 | 381.92 ± 88.61 | 439.78 ± 100.68 |
| Total cholesterol | 189.69 ± 53.81 | 216 ± 45.60 | 210.78 ± 72.14 | 187.61 ± 43.86 |
| Total hyperlipemia | 0.65 ± 0.48 | 0.40 ± 0.54 | 0.78 ± 0.42 | 0.61 ± 0.50 |
| Triglycerides | 150 ± 95.38 | 108.6 ± 30.22 | 194.5 ± 151.19 | 179.94 ± 147.16 |
| TSS Total | 2.60 ± 1.85 | 0.2 ± 0.44 | 4.57 ± 2.73 | 0.05 ± 0.23 |
| TST | 379.33 ± 96.45 | 382.20 ± 94.76 | 334.07 ± 84.01 | 381.64 ± 107.08 |
| Urea | 30.93 ± 9.97 | 28 ± 11.09 | 29.64 ± 9.76 | 23.78 ± 8.79 |
| Uric acid | 5.34 ± 1.33 | 5.06 ± 1.15 | 5.32 ± 1.19 | 4.80 ± 1.58 |
| VA | 94.40 ± 15.57 | 93.80 ± 3.96 | 79.57 ± 15.82 | 100.50 ± 10.48 |
| VCM | 90.12 ± 4.49 | 92 ± 2.30 | 149.49 ± 221.78 | 89.82 ± 3.68 |
| Waist circumference | 105.04 ± 11.14 | 99 ± 11.85 | 108.78 ± 12.09 | 99.16 ± 14.44 |
| WASO | 43.47 ± 28.16 | 35.20 ± 20.36 | 44.38 ± 17.14 | 49.60 ± 17.46 |
| Weeks since Dx | 16.78 ± 4.11 | 15.60 ± 4.15 | 15.35 ± 2.34 | 16.44 ± 4.28 |
| Weight | 85.94 ± 15.25 | 78.72 ± 18.97 | 82.60 ± 11.53 | 85.06 ± 17.90 |
| Z drugs | 0.26 ± 0.44 | 0.20 ± 0.44 | 0.14 ± 0.36 | 0.05 ± 0.23 |

**Abbreviation list:** %: Percentage. 6MWT: six-minute walk test. CT: computer tomography. DLCOc: diffusing capacity of the lungs for carbon monoxide. HDL: High-density lipoprotein. TG: triglyceride. ACE/AII: ACE inhibitors and AII receptor antagonists. ARDS: Acute respiratory distress syndrome. BNM: Neuromuscular Blockade. CHCM: mean corpuscular hemoglobin concentration. CK: creatine kinase. HrQOL: Health-related quality of life. CHD: Coronary heart disease. CKD: Chronic kidney disease. HFNC: High flow nasal cannula (CNAF). COPD: Chronic obstructive pulmonary disease. CXCL: chemokine (C-X-C motif) ligand (CXCL) family. ICU: intensive care unit. IMV: Invasive mechanical ventilation. DBP: Diastolic blood pressure. Spo2: Saturation of Peripheral Oxygen. ESS: Epworth Sleepiness Scale Sleep Test Questionnaire. FEV1: Forced expiratory volume in 1 second. GGT: Gamma-glutamyl transferase. GOT (AST): Glutamic oxaloacetic transaminase. GPT (ALT): Alanine Aminotransferase. HADS-A: Hospital Anxiety and Depression Scale-HADS Anxiety. HADS-D: Hospital Anxiety and Depression Scale-HADS Depression. HCM: mean corpuscular hemoglobin. HCO3: Bicarbonate. HDL: High-density lipoprotein cholesterol. HTA: Arterial hypertension. HOMA: Homeostatic model assessment. HR: Heart Rate. ISI: Insomnia Severity Index. IRSS: Selective serotonin reuptake inhibitors. Ig: Immunoglobulins. IL: Interleukin. LDH: Lactate Dehydrogenase. LDL: Low-density lipoprotein cholesterol. MEQ: The Morning Evening Questionnaire. mMRC: Modified Medical Research Council Dyspnea Scale. NAFLD: Nonalcoholic fatty liver disease. O2. Oxygen. ODI: Oxygen desaturation index. Pafi: PaO2/FiO2 ratio. pAO2: Partial pressure of oxygen in the arterial blood. pCO2: Partial pressure of carbon dioxide. PCR: Polymerase chain reaction. PEF: Peak expiratory flow. pH: Potential of hydrogen. pO2: Partial pressure of oxygen. EuroQoL: European Quality of Life. FEF: Forced mid-expiratory flow. FEV1: Forced expiratory volume in 1 second. FVC: Forced vital capacity. VEF1: Forced expiratory volume in 1 second. RDI: Respiratory Disturbance Index. A/G: Albumin/globulin. SATED: Satisfaction. Alertness. Timing. Efficiency and Duration Questionnaire for Sleep Health Measurement. Smet: Structured Multidisciplinary Work Evaluation Tool. SBP: Systolic blood pressure. DBP: Diastolic blood pressure. SD: Standard deviation. SF: Short Form. SQALI: Sleep Apnea Quality of Life Index. Stop Bang: The snoring. tiredness. observed apnea. high BP. BMI. age. neck circumference. and male gender. T90: Percentage of time spent in sleep below 90% oxygen saturation. TNF-a: Tumor Necrosis Factor alpha. TNS: Total night sleep. TSS: Total severity score. TST: Percentage of total sleep time. VCM: Mean corpuscular volume. WASO: Wakefulness after sleep onset.

**Supplementary Table 2:** Classification models for Random Forest and XGBoost.

| **Model** | **Class** | **Accuracy** | **Precision** | **Recall** | **F1-score** |
| --- | --- | --- | --- | --- | --- |
| Random Forest | Normal | 1.00 | 1.00 | 1.00 | 1.00 |
|  | DLCOc | 1.00 | 1.00 | 1.00 | 1.00 |
|  | CT | 0.80 | 1.00 | 0.80 | 0.89 |
|  | CT+DLCOc | 1.00 | 0.60 | 1.00 | 0.75 |
| XGBoost | Normal | 1.00 | 1.00 | 1.00 | 1.00 |
|  | DLCOc | 1.00 | 0.89 | 1.00 | 0.94 |
|  | CT | 1.00 | 1.00 | 1.00 | 1.00 |
|  | CT+DLCOc | 0.67 | 1.00 | 0.67 | 0.80 |

**Supplementary Table 3.** Clinical characteristic of proteomic COVID-19 subgroups during acute phase (n=16).

|  | **CT**  **n=8** | **CT and DLCOc**  **n=8** |
| --- | --- | --- |
| **Gender male:female N (%)** | 5:3  (62.5:37.5) | 4:4  (50:50) |
| **Age (years). (SD)** | 51.4 ± 8.6 | 57.5 ± 8.0 |
| **ABO Group** |  |  |
| A. N (%) | 2 (25) | 3 (37.5) |
| B. N (%) | 1 (12.5) | 2 (25) |
| AB. N (%) | 1 (12.5) | 0 (0) |
| O. N (%) | 4 (50) | 3 (37.5) |
| **Tobacco status** |  |  |
| Current. N (%) | 2 (25) | 1 (12.5) |
| Former. N (%) | 1 (12.5) | 2 (25) |
| Never Smoker. N (%) | 5 (62.5) | 5 (62.5) |
| **Alcohol usage** |  |  |
| Never. N (%) | 2 (25) | 4 (50) |
| Occasionally. N (%) | 6 (75) | 2 (25) |
| Frequently. N (%) | 0 (0) | 2 (25) |
| **COVID-19 severity** |  |  |
| Mild. N (%) | 0 (0) | 0 (0) |
| Moderate. N (%) | 0 (0) | 1 (12.5) |
| Severe/critical. N (%) | 8 (100) | 7 (87.5) |
| ARDS. N (%) | 8 (100) | 8 (100) |
| **Comorbidities** |  |  |
| Arterial hypertension. N (%) | 4/8 (50) | 5/8 (62.5) |
| IR at baseline. N (%) | 1/8 (12.5) | 4/8 (50) |
| T2DM at baseline. N (%) | 1/8 (12.5) | 1/8 (12.5) |
| Heart Failure. N (%) | 0 (0) | 0 (0) |
| COPD | 0 (0) | 0 (0) |
| Cancer | 0 (0) | 0 (0) |
| CKD | 0 (0) | 0 (0) |
| Afib | 0 (0) | 0 (0) |
| Stroke | 0 (0) | 1/8 (12.5) |
| CHD | 0 (0) | 0 (0) |
| NAFLD | 1/8 (12.5) | 3/8 (37.5) |
| Hypothyroidism | 1/8 (12.5) | 2/8 (25) |
| **Acute**  **COVID-19 parameters: Lab test** |  |  |
| Ferritin  (mg/dL). (SD) | 1914 ±1294 | 2173 ± 1442 |
| C-Reactive Protein  (mg/dL). (SD) | 141.6 ± 113.1 | 168 ± 126.0 |
| White blood cell count  (x10^9^/L). (SD) | 13203 ± 5660 | 10831 ± 5786 |
| Lymphocyte cell count  (x10^9^/L). (SD) | 1026 ± 335.1 | 771.9 ± 325.6 |
| Lymphocyte from WBC (%) | 7.8 | 7.1 |
| D-Dimer  (mg/dL). (SD) | 2589 ± 1513 | 1389 ± 1109 |
| Fibrinogen (mg/dl). (SD) | 640.7 ± 157.8 | 876.2 ± 416.1 |
| PaO2/FIO2 ratio. (SD) | 167.0 ± 29.8 | 160.9 ± 34.6 |
| **Acute**  **COVID-19 parameters: Medical** |  |  |
| Days in hospital (days). (SD) | 37.60 ± 30.85 | 33.86 ± 25.63 |
| Days in intensive care unit (days). (SD) | 15.63 ± 9.02 | 14.5 ± 9.8 |
| Days in invasive mechanical ventilation (days). (SD) | 12.75 ± 9.39 | 11.50 ± 7.52 |
| Invasive mechanical ventilation N. (%) | 8/8 (100) | 7/8 (87.5) |
| High Flow Nasal Cannula N. (%) | 0/8 (0) | 6/8 (75)** |
| Awakening prone position N. (%) | 1/8 (12.5) | 5/8 (62.5)* |
| **Acute**  **COVID-19 parameters: Therapy** |  |  |
| Steroids N. (%) | 3/8 (37.5) | 7/8 (87.5)* |
| Biologic Therapy N. (%) | 0/8 (0) | 0/8 (0) |
| Multinodular goiter N. (%) | 6/8 (75) | 6/8 (75) |
| Prono N. (%) | 5/8 (62.5) | 4/8 (50) |
| Tracheostomy N. (%) | 3/8 (37.5) | 3/8 (37.5) |
| **Symptoms during acute phase** |  |  |
| Fever. N (%) | 6/8 (75) | 5/8 (62.5) |
| Headache. N (%) | 4/8 (50) | 4/8 (50) |
| Chest pain. N (%) | 5/8 (62.5) | 5/8 (62.5) |
| Sore throat. N (%) | 4/8 (50) | 3/8 (37.5) |
| Cough. N (%) | 4/8 (50) | 5/8 (62.5) |
| Dyspnea. N (%) | 7/8 (87.5) | 8/0 (100) |
| Polypnea. N (%) | 6/8 (75) | 6/8 (75) |
| Myalgia. N (%) | 5/8 (62.5) | 3/8 (37.5) |
| Desaturation. N (%) | 0/8 (0) | 0/8 (0) |
| Abdominal Pain. N (%) | 2/8 (25) | 0/8 (0) |
| Diarrhea. N (%) | 3/8 (37.5) | 0/8 (0) |
| Change smell. N (%) | 3/8 (37.5) | 3/8 (37.5) |
| Change taste. N (%) | 3/8 (37.5) | 2/8 (25) |

**Abbreviation list:** SD: Standard deviation. IR: Insulin resistance. T2DM: Type 2 diabetes mellitus. PaO_2_/FIO_2_: the partial pressure of oxygen in the arterial blood/ the fraction of inspired oxygen. COPD: Chronic obstructive pulmonary disease. CKD: Chronic kidney disease. Afib: atrial fibrillation. CHD: Coronary heart disease. NAFLD: Non-alcoholic fatty liver disease.

**Supplementary Table 5. Clinical characteristic of the patients with cardiac infarction (n=25)**

| Patient Code | **Age** | **Gender** | **Diagnostic** | **Location** |
| --- | --- | --- | --- | --- |
| **LOX-1** | 40 | Male | STEMI | Arterial wall |
| **LOX-2** | 54 | Male | STEMI | Arterial wall |
| **LOX-3** | 58 | Male | STEMI | K2 |
| **LOX-4** | 45 | Male | STEMI | Inferior wall |
| **LOX-5** | 80 | Female | STEMI | Inferior wall |
| **LOX-6** | 64 | Male | STEMI | Posterior wall |
| **LOX-7** | 93 | Male | STEMI | Inferior wall Atrioventricular block |
| **LOX-8** | 64 | Male | STEMI | Posterior wall |
| **LOX-9** | 50 | Male | STEMI | Anterior wall |
| **LOX-10** | 49 | Male | STEMI | Inferior wall |
| **LOX-11** | 74 | Male | STEMI | Inferior wall |
| **LOX-12** | 49 | Male | STEMI | Inferior wall |
| **LOX-13** | 77 | Female | STEMI | Extensive anterior |
| **LOX-14** | 63 | Male | STEMI | Anterior wall |
| **LOX-15** | 70 | Male | STEMI | Anteroseptal |
| **LOX-16** | 62 | Male | STEMI | Inferior wall |
| **LOX-17** | 68 | Male | STEMI | Extensive anterior |
| **LOX-18** | 58 | Male | STEMI | Extensive anterior |
| **LOX-19** | 53 | Female | STEMI | Anteroseptal |
| **LOX-20** | 86 | Female | STEMI | Inferior wall |
| **LOX-21** | 52 | Male | STEMI | Inferior wall |
| **LOX-22** | 59 | Male | STEMI | Inferior wall posterior-lateral |
| **LOX-23** | 47 | Male | STEMI | Inferior- lateral wall |
| **LOX-24** | 53 | Male | STEMI | Inferior wall |
| **LOX-25** | 52 | Female | STEMI | Extensive anterior |

**Abbreviation list:** ST-segment elevation myocardial infarction (STEMI).
